# Diversity in Metabolites and Fruit Quality Traits in Blueberry Enables Ploidy and Species Differentiation and Establishes a Strategy for Future Genetic Studies

**DOI:** 10.3389/fpls.2020.00370

**Published:** 2020-04-03

**Authors:** Molla F. Mengist, Mary H. Grace, Jia Xiong, Colin D. Kay, Nahla Bassil, Kim Hummer, Mario G. Ferruzzi, Mary Ann Lila, Massimo Iorizzo

**Affiliations:** ^1^Plants for Human Health Institute, North Carolina State University, NCRC, Kannapolis, NC, United States; ^2^USDA-ARS-National Clonal Germplasm Repository, Corvallis, OR, United States; ^3^Department of Horticultural Science, North Carolina State University, Raleigh, NC, United States

**Keywords:** blueberry, health promoting phytochemicals, fruit size, ploidy, fruit quality, flavonoid pathway

## Abstract

Blueberry is well recognized as a rich source of health promoting phytochemicals such as flavonoids and phenolic acids. Multiple studies in blueberry and other crops indicated that flavonoids and phenolic acids function as bioactive compounds in the human body promoting multiple health effects. Despite their importance, information is limited about the levels of variation in bioactive compounds within and between ploidy level and species, and their association with fruit quality traits. Such information is crucial to define a strategy to study the genetic mechanisms controlling these traits and to select for these traits in blueberry breeding programs. Here we evaluated 33 health related phytochemicals belonging to four major groups of flavonoids and phenolic acids across 128 blueberry accessions over two years together with fruit quality traits, including fruit weight, titratable acidity, total soluble acids and pH. Highly significant variation between accessions, years, and accession by year interaction were identified for most of the traits. Cluster analysis grouped phytochemicals by their functional structure (e.g., anthocyanins, flavanols, flavonols, and phenolic acids). Multivariate analysis of the traits resulted in separation of diploid, tetraploid and hexaploid accessions. Broad sense heritability of the traits estimated in 100 tetraploid accessions, ranged from 20 to 90%, with most traits revealing moderate to high broad sense heritability (H^2^ > 40%), suggesting that strong genetic factors control these traits. Fruit size can be estimated as a proxy of fruit weight or volume and vice versa, and it was negatively correlated with content of most of phytochemicals evaluated here. However, size-independent variation for anthocyanin content and profile (e.g., acylated vs. non-acylated anthocyanin) exists in the tetraploid accessions and can be explored to identify other factors such as genes related to the biosynthetic pathway that control this trait. This result also suggests that metabolite concentrations and fruit size, to a certain degree can be improved simultaneously in breeding programs. Overall, the results of this study provide a framework to uncover the genetic basis of bioactive compounds and fruit quality traits and will be useful to advance blueberry-breeding programs focusing on integrating these traits.

## Introduction

Blueberry belongs to the Ericaceae family and the genus *Vaccinium* section Cyanococcus. Three blueberry species, that include the highbush blueberry (HB), *V. corymbosum* L., rabbiteye blueberry (RB), *V. ashei* Reade (syn. *Vaccinium virgatum* Ait) and native stands of lowbush blueberry (LB), *V. angustifolium* Ait, are commercially grown in the United States (Kalt et al., 2001; Lyrene et al., 2003; Hancock et al., 2008; Retamales and Hancock, 2018). Among these species, the HB blueberry is widely grown in the US, accounting for ∼95% of total blueberry production (Kalt et al., 2001; Wang et al., 2019). The HB cultivars are further classified into northern highbush (NHB) and southern highbush (SHB) blueberries based on chilling requirement and winter hardiness (Lyrene et al., 2003; Hancock et al., 2008; Retamales and Hancock, 2018). In addition to these species, a number of wild diploid blueberry species have been described and are used in breeding programs as sources for low chilling requirement, disease resistance (mummy berry disease), heat tolerance and adaptation to higher soil pH (Hancock et al., 2008; Lobos and Hancock, 2015; Retamales and Hancock, 2018; Wang et al., 2019). Studies have also focused on diploid wild blueberries as important sources of bioactive metabolites including flavonoids and phenolic acids (Kalt et al., 2001; Giovanelli and Buratti, 2009; Prencipe et al., 2014; Zoratti et al., 2015; Wang et al., 2019).

During the last decade, blueberry production and consumption have increased (Rodriguez-Saona et al., 2019) and the growing body of research supporting the health benefits associated with blueberry consumption have likely contributed to this growth (Norberto et al., 2013; Sun et al., 2019). Indeed, clinical evidence suggests that sufficient intake of blueberries provides multiple health benefits including lowering blood pressure, protecting against heart attack, preventing cancer, improving mental health and managing diabetes (Martineau et al., 2006; Krikorian et al., 2010; Stull et al., 2010; Norberto et al., 2013; Sun et al., 2019; Yang H. et al., 2019).

Important health–promoting bioactive metabolites, including flavonoids (anthocyanins, flavanols, and flavonols) and non-flavonoids such as phenolic acids, are found abundantly in blueberry (Kalt et al., 2001; Rodriguez-Mateos et al., 2012; Yousef et al., 2013, 2014; Gündüz et al., 2015; Scalzo et al., 2015; Li et al., 2017; Timmers et al., 2017; Grace et al., 2019; Wang et al., 2019). In blueberry, anthocyanins are the most abundant flavonoids, and are derivative of anthocyanidins (aglycones) by the addition of sugar moieties. The most common anthocyanidins in blueberry are delphinidin (Dp), cyanidin (Cyn), petunidin (Pet), peonidin (Peo), and malvidin (Mv). The anthocyanidins can be conjugated with sugar moieties like arabinoside, glucosides, and galactoside via the C3 hydroxyl group in ring C, and form structurally different anthocyanins. The conjugation of anthocyanidins with sugar moieties can be further modified through acylation (Norberto et al., 2013; Yousef et al., 2014; Li et al., 2017; Grace et al., 2019; Wang et al., 2019). Various patterns of conjugated sugar moieties, with or without acylation, can affect the chemical properties of anthocyanins, such as stability and bioavailability. Acylation of anthocyanins, through the addition of acyl groups such as organic acids (cinnamic and aliphatic acids) via ester bonds, improves color and tinctorial strength and increases the stability of anthocyanins at higher pH or under intense light and heat stress (Giusti and Wrolstad, 2003; Matera et al., 2015; Zhao et al., 2017; Strauch et al., 2019). Acylated anthocyanins are of special interest for use as a stable natural colorants in food industries (Giusti and Wrolstad, 2003; Matera et al., 2015; Zhao et al., 2017; Strauch et al., 2019).

Caffeic, ferulic, and chlorogenic acids are commonly reported phenolic acids. Of these, chlorogenic acid is the most abundant phenolic acid in blueberry (Grace et al., 2019; Wang et al., 2019). Similarly, flavanols (epi-catechin, catechin, proanthocyanidin B_1_, and proanthocyanidin B_2_) and flavonols (kaempferol, quercetin, and myricetin) are also commonly reported flavonoids in blueberry (Grace et al., 2019; Wang et al., 2019).

Differences in bioavailability between acylated and non-acylated anthocyanins have been reported in the literature (Kurilich et al., 2005; Charron et al., 2009; Oliveira et al., 2019). Charron et al. (2009) reported that non-acylated anthocyanins are better absorbed in the upper intestine compared to acylated anthocyanins. This effect may have been due, in part, to presence of phenolic acids as acylation agents on anthocyanins that serves to limit the transport efficiency or enzymatic conversion to aglycones in the upper intestine. While acylation does serve to stabilize anthocyanins to digestive conditions (McDougall et al., 2007; Correa-Betanzo et al., 2014), this would potentially serve to enhance their retention during intestinal passage and make them more available for catabolism by human microbiota in the large intestine (Fleschhut et al., 2006). As such, understanding factors promoting differences in acylation patterns could serve to better predict efficiencies of absorption, extent of host and microbial metabolism and ultimate health protective properties of anthocyanin rich foods.

Numerous studies have been conducted to examine genotype- or species-specific metabolites and fruit quality variability in blueberry (Kalt et al., 2001; Rodriguez-Mateos et al., 2012; Yousef et al., 2013, 2014; Gündüz et al., 2015; Scalzo et al., 2015; Li et al., 2017; Timmers et al., 2017; Wang et al., 2019). However, these studies targeted a small number of genotypes/accessions and provided limited insight into species and ploidy diversity of metabolite profiles and fruit quality traits. In addition, there is limited information about the association between metabolites with quality traits including fruit weight/size, pH, total soluble solid (TSS) and titratable acidity (TA) (Kalt et al., 2001; Rodriguez-Mateos et al., 2012; Yousef et al., 2013, 2014; Gündüz et al., 2015; Li et al., 2017; Wang et al., 2019). Most importantly, no studies have yet identified potential strategy to perform genetic analysis for these bioactive metabolites.

In this study, we profiled metabolites (anthocyanins, phenolic acids, flavanols, and flavonols) and fruit quality traits (pH, TA, TSS, fruit weight) in 128 blueberry accessions across three ploidy levels (diploid, tetraploid, and hexaploid) over two years. The main objectives of this study were to: (1) assess variability among the blueberry accessions for different metabolites and fruit quality traits; (2) investigate the association among metabolites, and between metabolites and fruit quality traits; and (3) establish a strategy to study the genetic basis controlling metabolite accumulation in highbush blueberry.

## Materials and Methods

### Materials Collection and Preparation

A collection of 128 blueberry accession*s* was obtained from the National Clonal Germplasm Repository (NCGR), Corvallis, OR, United States. Information about these *Vaccinium* accessions, including name, collection sites, ploidy level and species is provided in Supplementary Table S1. The collection included 100 tetraploid (4×), 22 hexaploid (6×), and 6 diploid (2×) accessions, representing diversity in geographical origin and genetic background (Supplementary Table S1). Of the 100 tetraploid (4×) accessions, 98 accessions represent *V. corymbosum*, NHB, SHB, and hybrids between these two types of blueberry cultivars. Berries were harvested at ripening stage for two consecutive years (2017 and 2018). For each accession, fruit were harvested from two or three clones. Since amount of fruit available for each clone was not the same and in several case not sufficient to perform all the phenotyping assays, prior to processing, the fruits were combined and then separated into three technical replicates. The technical replicates could minimize errors associated with sample processing and fruit quality and metabolite traits phenotyping. After harvesting, the berries were stored at −80°C, shipped on dry ice to the Plants for Human Health Institute (PHHI), Kannapolis, North Carolina, United States, and stored at −80°C until processing. Frozen berries (approximately 10–30 g, three replicates), were then used for fruit quality and metabolite analyses.

### Fruit Quality Trait Evaluation

#### Establishment of Phenotyping Method for Fruit Size

Image-based phenotyping is a powerful tool to estimate fruit quality attributes including fruit shape, size and color (Diaz-Garcia et al., 2016). While this method is high-throughput, it is also relatively time-consuming (sample set up, image acquisition and processing) if we are interested in phenotyping a single trait. Preliminary data from our lab suggested that image-based fruit volume estimation using the GiNA R package (Diaz-Garcia et al., 2016) and fruit weight were highly correlated. This association provides an opportunity to phenotype fruit size using fruit weight as a proxy for estimating fruit size, a faster phenotyping method as compared to fruit volume measurement. To verify this hypothesis, we selected 54 accessions varying in fruit weight. A minimum of 10 berries for each accession were used to estimate the fruit weight (g per fruit) of each berry. The same berries were scanned with a digital camera and the images were processed using the GiNA R package (Diaz-Garcia et al., 2016), which measured fruit volume and fruit surface area of each berry. Correlation was performed between all the measured parameters, to determine the relationship. To assess the agreement between the two measurements, fruit volume (cm^3^) and fruit weight (g), Bland-Altman plot was created using excel (Bland and Altman, 1986). Bland-Altman plot determines the bias (mean of the differences) and limits of agreement [bias ± 2 × SD (standard deviation)].

#### Phenotyping of Fruit Quality Traits

Fruit weight (g per fruit) was recorded (10–30 berries, three replicates) for fruit harvested in 2017 and 2018. In addition to fruit weight, we evaluated TSS, pH and TA. The berries used to measure fruit weight were homogenized to a puree in a Waring Commercial Blender 7012G (Torrington, CT, United States). Homogenized samples were used to determine TSS, pH and TA and to quantify anthocyanins and non-anthocyanin bioactive metabolites using high-performance liquid chromatography (HPLC) and liquid chromatography–mass spectrometry (LC-MS).

Total soluble solid was estimated using a digital hand-held “pocket” refractometer PAL-1 (Atago, Tokyo, Japan) and the results were expressed as°Brix. pH and TA were measured using 1 g of homogenized sample diluted with 30 ml pre-boiled double distilled water. The pH was measured using Accumet AB15, pH-meter (Fisher Scientific, Waltham, MA, United States). Then, TA was determined with a Mettler DL15 Auto-Titrator (Columbus, OH, United States) at pH of 8.2 using 0.02 mol L^–1^ sodium hydroxide. TA was expressed as percentage of citric acid (wt/wt) per 1 g FW.

### Extraction and Quantification of Anthocyanins and Non-anthocyanin Metabolites

#### Extraction and Sample Preparation for HPLC and LC-MS Analysis

An aliquot (3 g) of the homogenized blueberry puree was weighed in a 30-mL centrifuge tube. After the addition of 8 ml of 80% methanol in water (containing 5% formic acid), this mix was homogenized using a PRO0250 (PRO Scientific Inc., Oxford, CT, United States) for 2 min to extract polyphenols. The homogenate was centrifuged (Sorvall RC-6 plus, Asheville, NC, United States) for 2 min at 4,000 rpm. The supernatant was collected in a 25-ml volumetric flask. The residue was then extracted two more times, once with 8 ml of the same solvent, and then with 100% methanol. Supernatants were collected and brought to a final volume of 25 ml. About 1 ml of each sample was diluted with equal volume of methanol-water-formic acid, 65:35:5 and filtered (0.22 μm PTFE membrane) prior to HPLC-PDA analysis for anthocyanins and chlorogenic acid.

For LC-MS analysis of other phenolic compounds of low concentrations (flavonols, flavanols, and phenolic acids), a solid phase extraction (SPE) procedure was performed to remove sugars that may cause decrease in sensitivity of the MS system. An aliquot of 1–1.5 mL from each sample/replicate was centrifuged at 15,000 rpm for 5 min. In a 96-deep well plate (loading plate), 1.2 mL water (1% formic acid, FA), 10 μL fisetin (200 μg/mL, final concentration, 5 μg/mL), and 200 μL extract were mixed. The SPE plate (Phenomenex; StrataTM 96 Well Plate 10 mg/Well) was preconditioned with methanol (1% FA), then with water (1% FA). The samples were transferred from the loading plate to the SPE plate and drained by gravity. The 96-SPE well plate was washed 5 times with 600 μL water (1% FA) and dried about 20 min using positive pressure manifold. Finally, the samples from the SPE plate were eluted with 300 μL methanol (0.1% FA) to a 96-well collection plate. Then, 90 μL water (0.1% FA) and 10 μL internal standard (phlorizin 200 μg/mL, final concentration 5 μg/mL) was added to each well. The collection plate was covered with a sealing mat and immediately transferred to the auto-sampler of the MS system for analysis.

#### HPLC Analysis

HPLC analysis was conducted to quantify anthocyanins and chlorogenic acid. Standards, cyanidin-3-galactoside, cyanidin-3-glucoside, and malvidin-3-galactoside, were obtained from Chromadex (Irvine, CA, United States). Delphinidin-3-glucoside was purchased from Cayman Chemicals (Ann Arbor, MI, United States). Delphinidin-3-galactoside, malvidin-3-glucoside, petunidin-3-glucoside, myricetin-3-glucoside, kaempferol-3-glucoside, and syringetin-3-glucoside were obtained from Extrasynthese (Genay Cedex, France). Cyanidin-3-arabinoside and peonidin-3-glucoside were obtained from Polyphenols (Sandnes, Norway).

Each of the nine anthocyanin reference compounds and chlorogenic acid standard were individually dissolved in methanol-water-formic acid, 65:35:5, at a concentration of 5 mg/mL. Equal volumes from each standard solution were mixed together and diluted with the solvent mix to prepare a standard stock mix solution (200 μg/mL). Eight standard working solutions, used for the calibration curve, were prepared by appropriate dilution of the stock mix solution (2–175 μg/mL). The reference standard mix dilutions were injected to generate an eight-point calibration curve for each compound, separately. Standard curves were linear with *R*^2^ > 0.9997 ± 0.0007.

The chromatography was conducted on an Agilent 1260 HPLC with diode array detector (DAD) (Agilent Technologies, Santa Clara, CA, United States). Separation of anthocyanins was performed on a Supelco C-18 column (25 cm × 4.6 mm × 5 μm), and the temperature of the column oven was maintained at 30°C. The eluents were water (formic acid 5%, v/v) (A) and methanol (B), with a gradient of 10–20% B (0–5 min), 20–25% B (5–20 min), 25–30% B (20–25 min), 30–35% B (25–30 min), 35–90% (30–43 min), and isocratic at 90% B (43–46 min). The column was then re-equilibrated for 4 min at 5% B, at the flow rate of 1 ml/min. Absorption was recorded at 520 nm for anthocyanins, and 280 nm for chlorogenic acid. Not all anthocyanins present in blueberry are commercially available; therefore, anthocyanins with no standard reference were quantified as their corresponding glucoside or galactoside equivalent. The lowest limit of detection (LLD) for all anthocyanins was in the range of 1.24–1.91 ppm, 0.96 ppm for chlorogenic acid (Supplementary Table S2).

#### LC-MS Analysis

LC-MS analysis was conducted to quantify non-anthocyanin compounds including flavonols, flavanols and phenolic acids. Standards, procyanidins B1 and B2, catechin, epicatechin, caffeic acid, 2,4-dihydroxybenzoic acid, quercetin glucoside and galactoside and quercetin arabinoside were purchased from Sigma-Aldrich (St. Louis, MO, United States). Phlorizin was used as an internal standard; Fesitin was used to measure the efficiency of SPE, and both were purchased from Sigma-Aldrich. The analysis was performed on a hybrid IT-TOF mass spectrometer (Shimadzu LC-MS-IT-TOF, Kyoto, Japan) equipped with two LC-20AD pumps, a SIL-20AC autosampler, a CTO-20A column oven, an SPD-M20A PDA detector, a CBM-20A system controller coupled to an IT-TOF-MS through an ESI interface. All data were processed by Shimadzu LCMS lab Solution Version 1.2. The mass spectrometer was programmed to carry out a full scan over *m/z* 70–100–700 (MS1) and *m/z* 70–500 (MS2) in the negative ionization mode. The heat block and curved desolvation line (CDL) temperature were maintained at 200°C; nitrogen was used as the nebulizing gas at a flow rate of 1.5 L/min, and as the drying gas at 75 kPa; the interface voltage was (+), 4.5 kV; (-), -3.5 kV; and the detector voltage was 1.80 kV.

The chromatography was performed on a Shim-pack XR-ODS column (50 mm × 3.0 mm × 2.2 μm) (Shimadzu, Japan), and the temperature of the column oven was maintained at 50°C. The eluents were water (FA 0.5%, v/v) (A) and methanol (B), with a gradient of 5–50% B (0–17 min), 50–80% B (17–18 min), and 80–5% B (18–19 min). The column was then re-equilibrated for 1 min at 5% B, at the flow rate of 0.6 mL/min. Compounds were quantified as their extracted-ion chromatograms (EIC) in the negative ion mode using phlorizin as an internal standard. The lowest limits of detection for all non-anthocyanin compounds ranged from 0.41 to 2.66 ppm as presented in Supplementary Table S2.

### Data Processing and Statistical Analyses

#### Analysis of Variance, Trait Heritability, and Correlation Analyses

To assess the magnitude of variation within and between ploidy groups, we computed a minimum, maximum and range of variation for all metabolites and fruit quality traits. Fold-change values were calculated independently for each metabolite and fruit quality trait, dividing the maximum value by the minimum value of each trait within ploidy group. To normalize metabolite data, a log2 transformation was applied on quantified values. Analysis of variance (ANOVA) was performed to partition individual metabolites and fruit quality related trait according to ploidy, accession number, year, and accession by year interaction. Best linear unbiased estimate (BLUE) data obtained from the linear model were used as the phenotypic values for all subsequent analyses. Broad-sense heritability (H^2^) was estimated using variance components calculated from the restricted maximum likelihood (REML), calculated as follows

$${\text{H}}^{2}=\frac{\delta_{\text{g}}^{2}}{\left(\delta_{\text{g}}^{2}+\frac{\delta_{\text{gy}}^{2}}{\text{y}}+\frac{\delta_{\text{e}}^{2}}{\text{ry}}\right)}$$

where, $\delta_{\text{g}}^{2}$, $\delta_{\text{gy}}^{2}$, and $\delta_{\text{e}}^{2}\,$ are variance components of accessions, [genotype x environment] interaction, and residual variations, respectively; y is the number of environments (number of years in this study, 2) and r is the number of replications (3).

Pearson Coefficient of Correlation was performed to find the relationship among traits and for the two-year data, independently. The correlation was visualized using the R package corrplot (Wei et al., 2017).

#### Multivariate Analysis of Metabolites and Fruit Quality Traits

BLUE data obtained from linear effects model were used as an input file for hierarchical clustering (HC), principal component analysis (PCA) and partial least square discriminant analysis (PLS-DA). HC combines similar individuals or variables into clusters and arranges these clusters into a hierarchy while PCA is a technique used to reduce dimensionality of the data by finding linear combinations (dimensions; in this case, the number of metabolite and fruit quality traits) of the data. HC was performed with the Spearman and Ward’s methods, and were visualized as a heatmap with a dendrogram using the heatmap.2 R package (Warnes et al., 2016). PCA was performed using the R package FactoMiner (Lê et al., 2008) as a non-supervised method to identify key traits with the largest effect on the overall variability and to evaluate the effect of genetic background on fruit quality and metabolite profiles among different accessions. PLS-DA (supervised version of PCA) was performed using metaboanalyst (Chong et al., 2018) to examine the classification of accessions based on ploidy groups/species and also identify key features using variable importance for the projection (VIP) > 1. The model fitness was evaluated using model accuracy, Q^2^ and R^2^ values of the model as described (Szymańska et al., 2012).

## Results

### Fruit Weight and Image-Based Volume Phenotyping Methods

**FIGURE 1:**
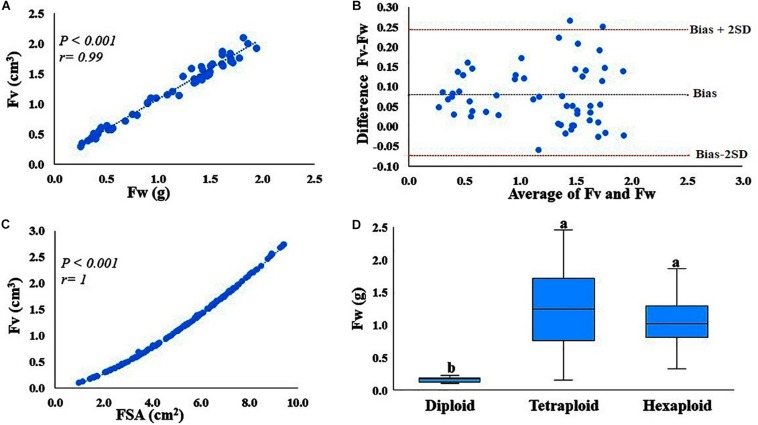
Correlation and agreement between fruit volume (Fv) and fruit weight (Fw), and Fw variations within and between ploidy level. **(A)** Correlation between fruit volume (cm^3^) measured using GiNA (image-based phenotyping) and fruit weight (g). **(B)** Agreement between the two measurements, Fv (cm^3^) estimated by GiNA and Fw (g). Bias is the mean of the differences between the two measurements, whereas bias-2SD and bias + 2SD are the lower and upper limits of the interval agreement, respectively. SD is the standard deviation of the mean of the differences. **(C)** Correlation between the two fruit parameters, fruit surface area (cm^2^) and fruit volume (cm^3^) as estimated by GiNA (image-based phenotying). **(D)** Fw variation between and within ploidy level.

Correlation analysis indicated that fruit weight and image-based volume measurements are highly (*P* < 0.001, *r* = 0.99) correlated (Figure 1A). Correlation provides information about the strength of the relationship between two measurements, but not agreement between the two measurements. Therefore, we assessed the agreement between the measurements, fruit volume (cm^3^) and fruit weight (g per fruit) with the Bland–Altman plot (Figure 1B). Here, the mean difference (bias) is 0.08, where the limits of agreement are −0.0734 and 0.25, indicating that 95% of the differences between the two measurements are within this range. Only one observation lies outside the 95% confidence interval, suggesting that the two measurements have an acceptable level of agreement, meaning that the absolute value of the numerical scale used to express the two measurements agree. Therefore, fruit weight can be used as a proxy to estimate fruit volume. It is also important to note that fruit surface area and fruit volume are also highly (*r* > 0.99) correlated traits (Figure 1C), suggesting that both fruit volume and fruit surface area can be highly predictable based on fruit weight. Furthermore, we also estimated fruit volume from 12 accessions with other fruit volume estimation methods including water displacement and texture analyzer and found that both methods are strongly (*r* > 0.99) correlated with fruit weight (Data not shown).

### Phenotypic Variability of Fruit Quality Traits

Phenotypic data for fruit quality traits including fruit weight, TA, pH and TSS, summarized by ploidy levels provided (Supplementary Table S3 and Supplementary Figure S1). The accessions evaluated here exhibited a considerable phenotypic variation for all traits, within and between ploidy-groups. For example, fruit weight exhibited ca. 2, 16- and 6-fold variation in diploid, tetraploid and hexaploid species, respectively (Figure 1D, Supplementary Table S3, and Supplementary Figure S1). TA had ca. 4, 9-, and 3-fold changes for diploid, tetraploid and hexaploid species, respectively. However, for pH and TSS, the variation among accessions was relatively low, less than two-fold changes for all ploidy groups (Supplementary Table S3 and Supplementary Figure S1). Overall, tetraploid accessions exhibited the highest level of variation for all fruit quality traits, probably due to the larger number of tetraploid samples evaluated in this study.

**FIGURE 2:**
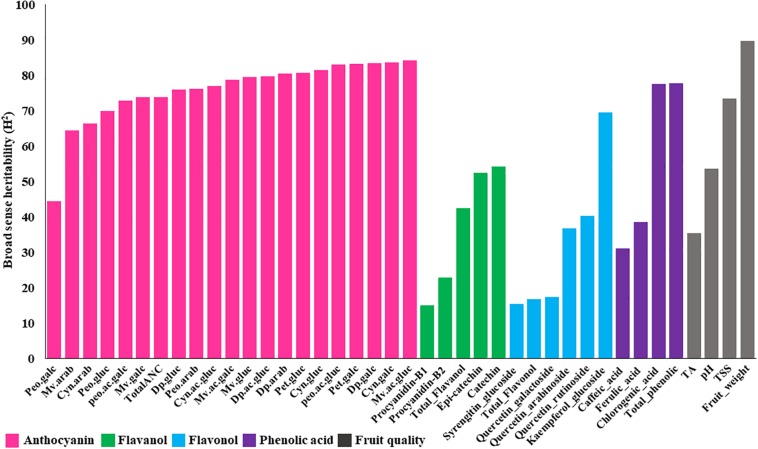
Broad sense heritability estimates for metabolites and fruit quality traits across 100 tetraploid accessions over two years. Anthocyanin abbreviations: Dp, delphinidin; Cyn, cyanidin; Peo, peonidin; Mv, malvidin; Pet, petunidin; gluc, -3-glucoside; galc, -3-galactoside; arab, -3-arabinoside; ac.gluc, -3-(6-acetyl glucoside); ac.galc, -3-(6-acetyl galactoside).

Combined analysis of variance showed significant (*P* < 0.01) effects for accession, year and accession by year interaction for all fruit quality traits except for pH, which did not show significant differences between years for any ploidy groups (Supplementary Table S4). Furthermore, broad sense heritability of fruit quality traits, estimated for tetraploid accessions (the largest group, *N* = 100), revealed a moderate to high (>40%) level of heritability (Figure 2). Fruit weight and TSS are highly heritable traits (>70%), suggesting these traits can be improved through phenotypic selection and that the genetic component may play a major effect on the observed variation.

### Phenotypic Variability in Metabolites

We identified 33 metabolites including 20 anthocyanins, 6 flavonols, 4 flavanols and 3 phenolic acid compounds. Of the 20 anthocyanins identified, 14 were non-acylated anthocyanins and 6 were acylated anthocyanins (Supplementary Table S2). We observed a high degree of variability for all metabolites, with ca. 2-, 5-, and 2-fold variations for total anthocyanin in diploid, tetraploid and hexaploid species, respectively (Supplementary Table S3). Individual anthocyanins such as peonidin-3-glucoside, peonidin-3-(6-acetyl) galactoside and malvidin-3-(6-acetyl) galactoside exhibited more than 17 fold variation within ploidy levels (Supplementary Table S3 and Supplementary Figure S1). Similarly, the degree of variability in total flavanol, total flavonol and total phenolic acid was examined between and within the different ploidy levels and it was found that tetraploid species had a higher degree of variability compared to diploid or hexaploid species (Supplementary Table S3 and Supplementary Figure S1). Overall, the degree of variability was higher in tetraploid accessions, suggesting this material represents a wider pool of genetic diversity and could be used to study the genetic basis of these metabolites.

Combined analysis of variance was performed independently at ploidy level. For tetraploid accessions, combined analysis of variance for metabolites showed significant (*P* < 0.01) effects for accession, year and accession by year interaction for all traits except for cyanidin-3-(6-acetyl glucoside), malvidin-3-(6-acetyl galactoside), peonidin-3-(6-acetyl glucoside), malvidin-3-(6-acetyl glucoside) and quercetin-3-rutinoside, which did not show significant differences between years (Supplementary Table S4). For hexaploid accessions, we observed significant (*P* < 0.01) effect of accession, year and accession by year for all traits except for delphinidin-3-arabinoside, peonidin-3-galactoside, malvidin-3-arabinoside, malvidin-3-(6-acetyl galactoside), catechin, quercetin-3-galactoside and kaempferol-7-glucoside, which did not exhibit significant year effects (Supplementary Table S4). Regarding diploid accessions, significant (*P* < 0.01) variations were exhibited among accessions for all metabolites except for delphinidin-3-glucoside and quercetin-3-rutinoside, while most of the metabolites were not significantly affected by year and year by accession interaction (Supplementary Table S4). The smaller sample size of the diploid accessions (*N* = 6) as compared to the hexaploid and tetraploid accessions, probably limited our ability to exploit the full spectrum of phenotypic variation (Supplementary Figure S1) for fruit quality and metabolite content naturally existing in this germplasm, and to determine year or year × accession interaction effects (Supplementary Table S4).

We also estimated the broad sense heritability of bioactive metabolite using tetraploid accessions. The results showed moderate to high (>40%) range of heritability for all anthocyanin metabolites (Figure 2), suggesting that a significant portion of these variations have a genetic basis. On the other hand, most of the flavanols, flavonols, and phenolic acids showed low heritability (<40%), suggesting that these traits may be highly influenced by environmental factors (Figure 2). Kaempferol-7-glucoside, catechin, epi-catechin, and chlorogenic acid exhibited moderate to high (>40%) broad sense heritability (Figure 2).

To compare the average composition of anthocyanidins relative to the total anthocyanins across different ploidy levels, means of the five-anthocyanidin classes, delphinidin, cyanidin, petunidin, peonidin, and malvidin, were calculated for each ploidy level. Regardless of ploidy levels, across all accessions malvidin and delphinidin were the two major anthocyanidin classes. Peonidin was the least abundant anthocyanin. Similarly, the flavanol, flavonol and phenolic acid profiles were compared for each ploidy level. For flavanol, the pattern of distribution is somewhat similar among the different ploidy levels. Catechin followed by procyanidin-B1 were the most abundant flavanols in blueberry whereas, epi-catechin and procyanidin-B2 were the least abundant flavanols (Supplementary Figure S2). Among phenolic acids, more than 95% of the total phenolic acids were represented by chlorogenic acid while caffeic acid and ferulic acid were detected at relatively low levels (Supplementary Figure S2).

To further examine the diversity of the different forms of anthocyanin for potential use in genetic studies we carried out a cluster analysis using the data from the tetraploid accessions. Cluster analysis identified two major anthocyanin clusters, one cluster containing the glucoside anthocyanins and the second cluster comprising the arabinoside and galactoside anthocyanins. Within each of these two clusters, acylated and non-acylated anthocyanins formed different sub-clusters. Another observation was that anthocyanidin aglycones clustered based upon their derivatives, petunidin clustered with malvidin, and delphinidin and peonidin clustered with cyanidin (Supplementary Figure S3).

### Correlation Between Bioactive Metabolites and Fruit Quality Traits

**FIGURE 3:**
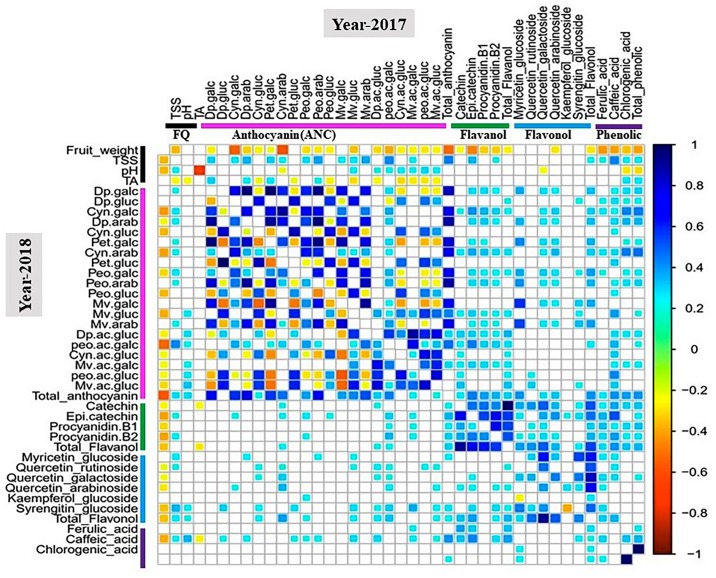
Correlation analysis of metabolite and fruit quality traits for 100 tetraploid accessions. White boxes represent non-significant correlations. The color bar indicates metabolite classes and fruit quality traits. Cyan-to-blue and yellow-to-red colors show significant (*P* < 0.05) positive and negative correlation between traits, respectively. Abbreviation: FQ, fruit quality trait.

Pearson coefficient of correlation analysis between metabolite and fruit quality traits was performed for all tetraploid and hexaploid accessions (Figure 3 and Supplementary Figures S4, S5).

For the 2017 tetraploid phenotypic data, fruit weight was negatively (*P* < 0.05) correlated with TSS, anthocyanins, flavanols, and phenolic acids. Smaller-sized fruits tended to have greater anthocyanin, flavanol and phenolic acids concentrations than the larger-sized fruits. However, fruit weight did not show any significant correlation with flavonols except for quercetin-3-arabinoside (Figure 3). In contrast, TSS was positively and significantly (*P* < 0.05) correlated with most of the anthocyanins, flavanols, and phenolic aids. As expected, TA showed a significant (*P* < 0.05) negative correlation with pH. However, pH did not exhibit any significant correlation with TSS or fruit weight (Figure 3). Another interesting observation was that pH was positively associated with acylated anthocyanins (Figure 3). Higher-pH accessions tended to exhibit greater acylated anthocyanin concentrations than the lower-pH accessions. These results suggest that acylation could be a pH dependent process. Similar correlation patterns were established for the 2018 phenotypic data (Figure 3).

For hexaploid accessions, there were no significant correlations between fruit weight and TSS, anthocyanins, flavonols, flavanols, and phenolic acids except for petunidin-3-arabinoside. Furthermore, TSS did not show any correlations with other fruit quality traits, anthocyanins, and flavanols (Supplementary Figure S4). The patterns of correlations among traits in tetraploid and hexaploid accessions were different. Fruit quality traits, fruit weight and TSS, showed significant associations with most of the metabolites in tetraploid accessions, but the same relationship was not observed in the hexaploid accessions. For the diploid accessions, the results highlighted that fruit weight was negatively (*P* < 0.05) correlated with chlorogenic acid and phenolic acids for both years, while for total anthocyanin fruit weight showed a significant (*P* < 0.05) negative correlation only for the 2017 phenotypic data (Supplementary Figure S5).

To identify tetraploid accessions that have a fruit size and anthocyanin content larger and higher than the average, we examined the relationship between anthocyanin concentration and fruit size based on the BLUE data. Accessions separated into four quadrants each representing the following phenotypes: (I) anthocyanin content higher than average and fruit size smaller than the average; (II) anthocyanin content and fruit size lower and smaller than the average; (III) anthocyanin content lower than the average and fruit size larger than the average; (IV) anthocyanin content and fruit size higher and larger than the average. As expected, a large number of accessions with high anthocyanin content and small fruit size were in quadrant I. However, four accessions with a fruit size and anthocyanin content larger than the average, were identified (Supplementary Figure S6).

### Multivariate Analysis of Metabolites and Fruit Quality Traits

**FIGURE 4:**
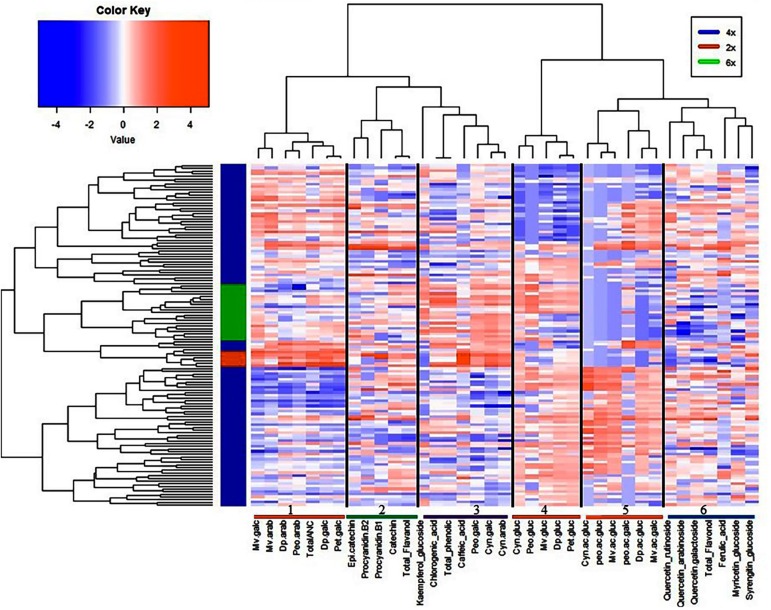
Hierarchical clustering analysis of metabolites and fruit quality traits from all 128 accessions. Six clusters represent the different metabolite classes, anthocyanins (1, 4, 5); flavanols (2); phenolic acids (3); and flavonols (6).

To provide an insight into the relationships/similarity among the different metabolites, a hierarchical clustering (HC) analysis was performed. The metabolites grouped largely into six major clusters (Figure 4), representing flavanols (cluster 2), flavonols (cluster 6), phenolic acids (cluster 3), and three clusters (1, 4, 5) representing the 20 anthocyanins (Figure 4). The three clusters of anthocyanins represent glucoside vs. galactoside/arabinoside anthocyanin (cluster 1 and 4, respectively) and acylated anthocyanins (cluster 5) (Figure 4).

Following HC analysis, we examined the data using PCA. The scree plot of the PCA demonstrated the variance explained for each component. Accordingly, the first five principal components (PCs) accounted for 63.8% of the variance (Supplementary Figure S7). Among these, the first three PCs accounted for 22.9, 17.3, and 12.8% of the variance, respectively (Supplementary Figure S7). To identify the key traits discriminating the different accessions, loading scores of the first two PCs were examined (Supplementary Figure S8) and metabolites with highest loading scores on PC1/2 were identified for all 128 accessions (Supplementary Figure S9A). Metabolites that contain galactoside sugar moieties (petunidin-3-galactoside, delphinidin-3-galactoside, cyanidin-3-galactoside and peonidin-3-galactoside) followed by arabinoside anthocyanins (cyanidin-3-arabinoside, cyanidin-3-arabinoside, delphinidin-3-arabinoisde and peonidin-3-arabinoside) showed the highest loading score in PC1. Whereas variables such as malvidin-3-(6-acetyl glucoside), malvinidin-3-glcuoside, total flavanol, delphinidin-3-(6-acetyl glucoside), malvidin-3-(6-acetyl glucoside), peonidin-3-(6-acetyl glucoside) and fruit weight explained most of the variability of the accessions in PC2 (Supplementary Figure S9B). A PCA biplot (Supplementary Figure S10) demonstrates the relationship between individuals and variables. For example, accessions PI554796, PI618230 and PI296406, and PI346623, PI554795 and PI267851 had high scores for the acylated anthocyanins and total anthocyanin concentrations, respectively. In contrast, accessions PI618099 and PI618193 had high fruit weight.

**FIGURE 5:**
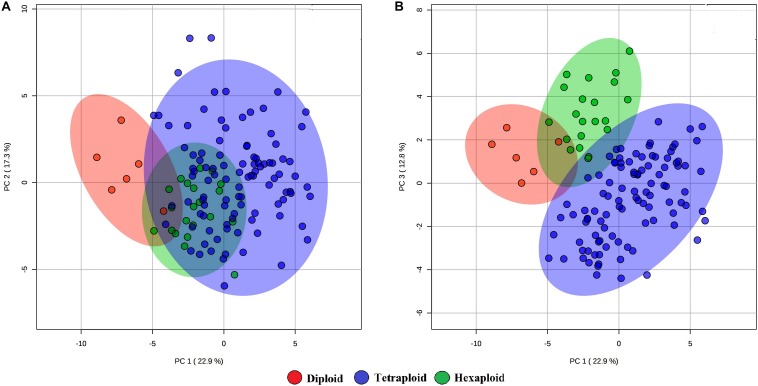
PCA score plots of the first and second **(A)** and first and third **(B)** principal components. The data obtained from metabolites and fruit quality data of 128 blueberry accessions across three ploidy levels (diploid, tetraploid, and hexaploid).

**FIGURE 6:**
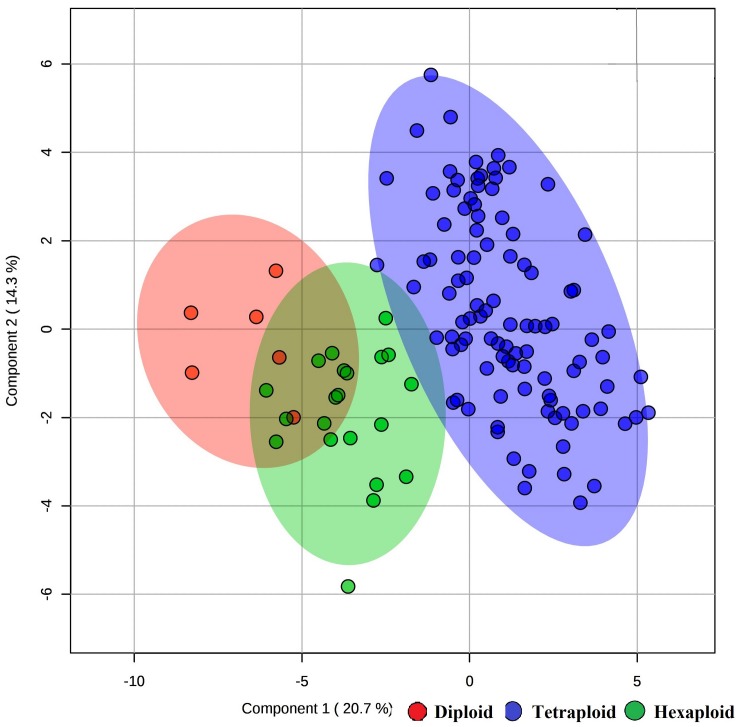
PLS-DA model of metabolite and fruit quality data from 128 blueberry accessions in three ploidy levels (diploid, tetraploid, and hexaploid). The figure representing the first two components of the PLS-DA model.

We examined whether metabolites and fruit quality data could discriminate accessions based on ploidy levels. PCA analysis revealed that diploid accessions clustered in a distinct group from other accessions (tetraploid and hexaploid), though no definitive separation was observed between hexaploid and tetraploid accessions for the first two PCs (Figure 5A). However, the first and third PCs of the PCA were able to separate all accessions by ploidy levels (Figure 5B). A supervised analysis, PLS-DA was applied to highlight separation of the accessions by ploidy levels and to identify key traits contributing to this separation. PLS-DA model on the first two PCs (accuracy = 0.95, *R*^2^ = 0.75, and *Q*^2^ = 0.70), separated accessions into their respective ploidy levels (Figure 6). Examination of variable importance projection (VIP > 1) suggests that the variables cyanidin-3-galactoside, delphinidin-3-(6-acetyl glucoside) and TSS significantly contributed to separate the accessions based on ploidy level (Supplementary Figure S11).

**FIGURE 7:**
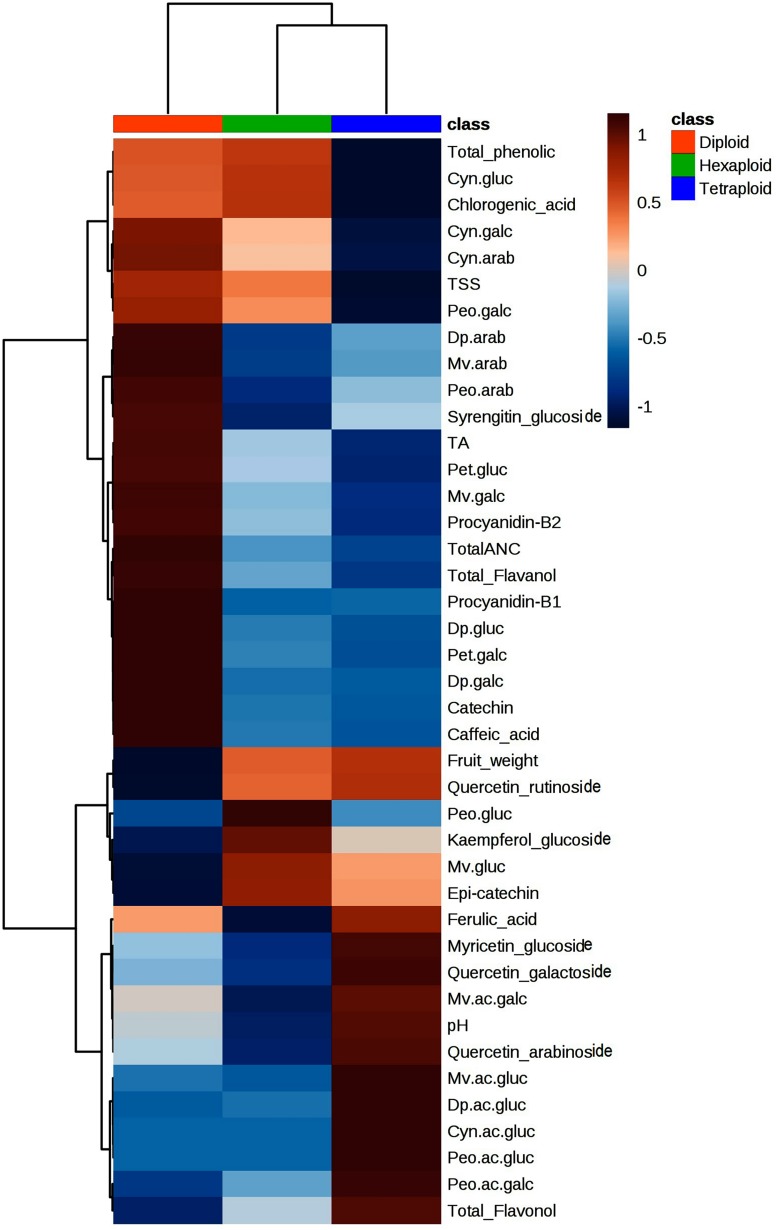
Heatmap showing variation of metabolites and fruit quality traits across the three-ploidy levels. Red and blue colors indicate highest and lowest performance of the traits, respectively.

We compared the metabolite concentrations and fruit quality traits across ploidy levels and found that tetraploid accessions had higher pH, acylated anthocyanin and flavonols concentrations than diploid and hexaploid accessions (Figure 7). It is important to note that pH of tetraploid accession is positively associated with acylated anthocyanins (Figure 3). The higher pH of the tetraploid accessions may have resulted in higher acylated anthocyanins as opposed to diploid and hexaploid accessions. Non-acylated anthocyanins and flavanols content were higher in diploid than hexaploid or tetraploid accessions. In contrast, tetraploid accessions had low total phenolic acid content, as compared to diploid and hexaploid accessions. The level of phenolic acids was comparable between diploid and hexaploid groups (Figure 7).

**FIGURE 8:**
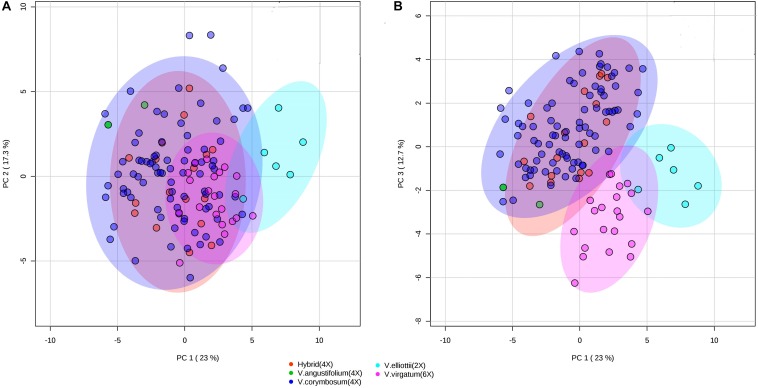
PCA score plots of the first and second **(A)** and first and third **(B)** principal components. The data obtained from metabolites and fruit quality data of 128 blueberry accessions from five species (hybrid, *V. elliottii*, *V. virgatum*, *V. angustifolium*, and *V. corymbosum*).

To assess whether the metabolites composition and fruit quality traits differentiate accessions by species, we performed PCA and PLS-DA. The PCA analysis revealed that *V. elliottii* formed a distinct group in the first two PCs. However, hexaploid and tetraploid species did not form a distinctive group (Figure 8A). Results from the first and third PCs of the PCA analysis revealed that *V. virgatum* and V. *elliottii* formed distinct groups while other species including tetraploid hybrids, *V. angustifolium* and *V. corymbosum* clustered as a single group (Figure 8). PLS-DA models also separated the accessions into three groups corresponding to *V. elliottii*, *V. virgatum* and other tetraploid (*V. corymbosum, V. angustifolium* and hybrids). Both PCA and PLS-DA analyses did not separate accessions of the same ploidy level into their respective species (Supplementary Figure S12). Furthermore, the key traits (cyanidin-3-galactoside, delphinidin-3-(6-acetyl glucoside) and TSS) identified, as most the discriminatory variables for the species analysis were the same variables that separated the accessions by ploidy by PLS-DA analysis (Supplementary Figure S11). Further, both PCA and PLS-DA analyses did not separate the accessions according to SHB and NHB types (Supplementary Figure S13). Similarly, when the geographical collection site was used as a classifier, the accessions did not show any distinct grouping pattern, though accessions from Maine showed a tendency to cluster together (Supplementary Figure S14). Overall, our data demonstrate that the accessions could be grouped according to ploidy group, but not type (NHB vs. SHB) or geographical origin.

## Discussion

Blueberry is recognized globally for its health promoting properties that have in part contributed to a rapid increase in demand and production over the past 15 years (Rodriguez-Saona et al., 2019). Multiple studies in blueberry and other crops indicated that polyphenols function as bioactive compounds in the human body promoting multiple health effects (Krikorian et al., 2010; Stull et al., 2010; Norberto et al., 2013). Despite their importance, limited research has been conducted in blueberry to characterize the extent of variation of metabolites in tetraploid cultivated germplasm, and their association with other fruit quality traits. Previous studies also used a limited number of cultivated accessions, and often used material that is not directly accessible to breeders and scientists (Kalt et al., 2001; Scalzo et al., 2013, 2015; Yousef et al., 2013, 2014; Gündüz et al., 2015; Timmers et al., 2017; Wang et al., 2019). In this study, we characterized the concentration of three major flavonoids and phenolic acids, and four fruit quality traits across a large set of tetraploid cultivated blueberry accessions (*N* = 100) and a number (*N* = 28) of other species with different ploidy. These plant materials represent clones of blueberry accessions publicly available through the USDA-NCGR and searchable through the Germplasm Resources Information – Global Network (GRIN-Global) database. The results were used to identify a strategy to perform a genetic study for bioactive metabolites in blueberry.

### Genotypic Effects Explain the Extensive Metabolite and Fruit Quality Traits Diversity Identified Within and Between Ploidy Groups

Extensive variation for concentrations and fruit quality traits was identified in this study, especially within the tetraploid accessions. Bioactive metabolite analysis identified 20 anthocyanins, 6 flavonols, 4 flavanols and 4 phenolic acids, in agreement with type and number of flavonoids and polyphenols observed in previous studies (Grace et al., 2019; Wang et al., 2019). We detected a pronounced accession, year and accession by year interaction effects on the metabolite concentrations. These interactions were expected since the metabolite concentration of a fruit crop, including blueberry, is governed by complex and interconnected enzymatic activities, each of which can react differently in response to the different growing environments (Chen et al., 2013; Wen et al., 2014; Matsuda et al., 2015; Colle et al., 2019). Similarly, there were significant effects of accession, year and year by environment interactions on fruit quality traits including fruit size, pH, TA and TSS. Similar results using a much smaller set of accessions were previously reported (Kalt et al., 2001; Yousef et al., 2014; Gündüz et al., 2015; Scalzo et al., 2015; Timmers et al., 2017).

**FIGURE 9:**
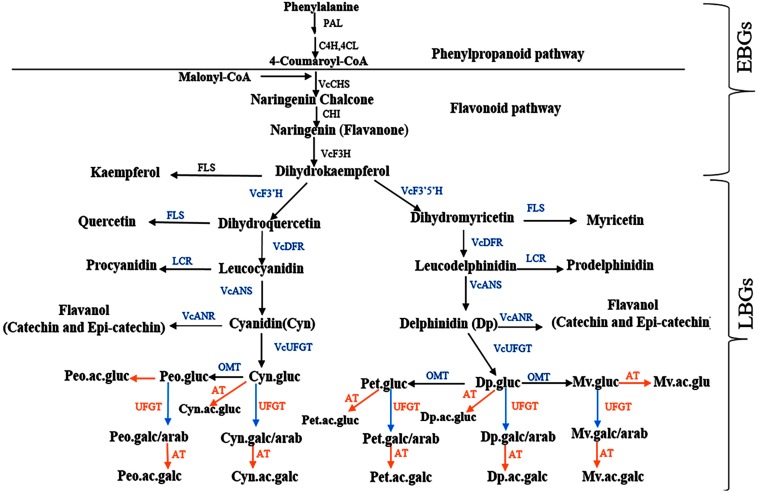
A schematic representation of anthocyanin pathway in blueberry with slight modification from bilberry as described in Jaakola et al. (2002). Enzyme abbreviations: C4H, Cinnamate 4-hydroxylase4; 4CL, 4-coumaroyl:CoA ligase; CHI, chalcone isomerase; F3′H, flavonoid 3′ hydroxylase; F3′5′H, flavonoid 3′5′ hydroxylase; LCR, leucoanthocyanidin reductase; UFGT, UDP Glc-flavonoid 3-*O*-glucosyl transferase; OMT,O-methyltransferase. Sugar and anthocyanidins abbreviations: gluc, glucoside; arab, arabinoside; galc, galactoside; Mv, malvidin; Pet, petunidin; and Peo, peonidin. Pathway genes: EBGs, early biosynthesis genes; LBGs, late biosynthesis genes.

Despite the significant accession by year interaction identified for most of the traits, the tetraploid accessions evaluated here, which are mostly cultivated and suitable for genetics studies, demonstrated moderate to high (>40%) broad sense heritability, indicating that genotypic effects explain most of the variability (Lourenço et al., 2017; Schmidt et al., 2019). To the best of our knowledge, this is the first study to estimate broad sense heritability for bioactive metabolites and fruit quality traits in blueberry and to provide preliminary insight for performing future genetic studies for these traits. The high level of broad sense heritability (>40%) detected here will help to improve QTL detection, especially in auto-polyploid species like blueberry (Bourke et al., 2019). Among the fruit quality traits, fruit weight had the highest broad sense heritability (>80%). Among the metabolites, the different forms of anthocyanins had the highest heritability followed by chlorogenic acid. As demonstrated by cluster analysis (Supplementary Figure S3), glycosylation and acylation, two enzymatic reactions that contribute to anthocyanin diversification in plants (Jaakola et al., 2002; Noguchi et al., 2009; Zifkin et al., 2012; Cheng et al., 2014; Le Roy et al., 2016) can explain the diversity observed within the tetraploid accessions. Previous studies described genes involved in flavonoid and more specifically anthocyanin biosynthesis in blueberry (Jaakola et al., 2002; Kurilich et al., 2005; Charron et al., 2009; Zifkin et al., 2012; Jaakola, 2013; Li et al., 2016; Lin et al., 2018; Colle et al., 2019). Here based on data from multiple studies (Jaakola et al., 2002; Zifkin et al., 2012; Jaakola, 2013; Colle et al., 2019), we reconstructed a scheme of the flavonoid pathway in blueberry (Figure 9) that includes early and late biosynthetic genes (EBGs and LBGs, respectively). The early anthocyanin genes (EBGs) are highly conserved across the plant kingdom including *Vaccinium* species (Jaakola et al., 2002; Zifkin et al., 2012; Jaakola, 2013; Li et al., 2016; Lin et al., 2018; Colle et al., 2019). However, the late anthocyanins biosynthesis genes (LBGs) have not fully explored in *Vaccinium*. Among the LBGs, the dihydroflavonol 4-reductase (DFR), anthocyanidin synthase (ANS), the (methyltransferase) OMT and a flavonoid 3-O-glucosyltransferase (*VcUFGT*) which play an important role into the diversification of the aglycone derivatives have been described (Jaakola et al., 2002; Zifkin et al., 2012). However, the LBGs regulating the downstream anthocyanin biosynthesis, including formation of galactoside, arabinoside, and the acylated derivatives, which contribute to the diversification of the anthocyanin, are still unknown. The results of this study highlight three aspects that can provide new insight in anthocyanin biosynthesis pathway in blueberry. First, the anthocyanidin aglycones were clustered based on their derivatives, petunidin and malvidin with delphinidin and peonidin with cyanidin (Figure 4 and Supplementary Figure S3), which suggest that probably a flux effect, toward each branch exist. These results are in agreement with the previous report in bilberry (Jaakola et al., 2002; Jaakola, 2013) and with the proposed pathway (Figure 9). Second, we observed clear clustering pattern between sugar moieties, glucoside vs. galactoside/arabinoside (Supplementary Figure S3). Previous study (Zifkin et al., 2012) reported that *Vc*UFGT gene product is involved in adding of glucoside on anthocyanidin structure in blueberry. However, no genes and genetic mechanisms have been described to regulate the formation of galactoside and arabinoside. Our cluster analysis suggests that one or more UFGT genes with major effect are most likely involved in catalyzing the synthesis of galactoside and/or arabinoside based anthocyanins. The third important point is that acylated anthocyanin also formed a subcluster (Supplementary Figure S3). Multiple families and types of acyltransferase (AT) have been described in plants (Giusti and Wrolstad, 2003; D’Auria, 2006; Matera et al., 2015) and could underlie the clear pattern of diversification observed here.

Studying the genetic mechanisms controlling anthocyanin diversification in blueberry is important since anthocyanin glycosylation contributes to anthocyanin stability and transport of anthocyanins to the vacuole (Jaakola et al., 2002; Noguchi et al., 2009; Zifkin et al., 2012; Jaakola, 2013; Cheng et al., 2014; Le Roy et al., 2016; Wang et al., 2018). Similarly, acylation contributes to stability under unfavorable conditions such as high pH, heat stress and intense light (Giusti and Wrolstad, 2003; D’Auria, 2006; Matera et al., 2015; Yang W. et al., 2019) and could be involved in bioaccessibility (Kurilich et al., 2005; Charron et al., 2009; Oliveira et al., 2019). Overall, the high broad sense heritability together with clear clustering patterns among the different anthocyanins based on sugar moieties and acylation suggest that key genetic factors/genes that have a relatively simple genetic inheritance are likely to be involved in the glycosylation and acylation of the different forms of anthocyanin in blueberry. Although this study does not identify specific genes involved in the anthocyanin diversification, the results provides a better understanding of the flux and underlying genetic effects controlling the downstream anthocyanin biosynthesis. This represent a solid framework for follow up genetic and functional analysis of this important pathway in blueberry. Similar levels of broad sense heritability were reported for metabolomes of diverse rice and maize accessions (Chen et al., 2013; Wen et al., 2014; Matsuda et al., 2015), or for fruit weight in cranberry (Georgi et al., 2013; Schlautman et al., 2015) and grape (Doligez et al., 2013), and were successfully used to dissect the genetic basis of these traits using genome-wide association analysis or QTL mapping.

Ploidy was the most distinctive descriptor to differentiate the germplasm evaluated here. Indeed, PCA and PLS-DA analyses clearly separated the three-ploidy groups (2×, 4×, and 6×). These results are consistent with previous studies reporting that HB (4×) blueberry differed from RB (6×) blueberry with respect to metabolite profile and fruit quality traits (Gündüz et al., 2015; Scalzo et al., 2015). Interestingly, traits with high level of heritability such as acylated anthocyanin, cyanidin derivatives, chlorogenic acid concentration or TSS had a high discriminatory importance, suggesting that a strong genetic footprint controlling these traits underlie the pattern of differentiation observed here across ploidy groups. In contrast, tetraploid species *V. corymbosum* and *V. angustifolium*, and blueberry types SHB and NHB were not differentiated, probably because they have a more uniform genetic makeup as compared to inter-ploidy groups. Indeed, hybridization between tetraploid NHB and LB is relatively common, as demonstrated by the high level of NHB and LB hybrids identified in invasive natural populations (Schepker and Kowarik, 1998), and their common use in breeding programs to develop the so-called tetraploid half-highbush cultivars like “Polaris,” “Chippewa,” “Northblue,” or “Northcountry” to cite a few examples (Bian et al., 2014). Similarly, NHB and SHB hybrids are very easy to obtain and are commonly used in breeding programs (Ballington, 2009; Hancock et al., 2018; Retamales and Hancock, 2018). Consistent with these observations, two independent studies (Campa and Ferreira, 2018; Zong et al., 2019) that used molecular markers to characterize blueberry germplasm revealed that the different ploidy (2×, 4×, 6×) were clearly differentiated, while SHB, NHB and half-highbush cultivars were not separated into different groups.

### Fruit Size Is a Key Factor to Study Genetic Mechanisms Controlling Flavonoids and Phenolic Acids in Blueberry

Our results demonstrated that not only fruit weight, surface area, and volume or size are very highly correlated (>0.99) but also that fruit weight and fruit volume measurements agreed. This information is relevant when planning phenotyping strategies to study the genetic mechanism controlling these traits. For instance, high-throughput phenotyping methods such as image analysis that can estimate fruit volume and number of fruits/plant, could be used to estimate fruit yield very accurately. Similarly, phenotyping instruments (e.g., texture analyzers) that integrate fruit weight measurements can be used to estimate fruit size.

Trait correlation analysis highlighted that in tetraploid accessions, fruit size was negatively correlated with anthocyanins, phenolic acids, flavanols and TSS content. Similar results for individual anthocyanins and total anthocyanin content, TSS and total phenolic acids were previously reported (Yousef et al., 2014; Gündüz et al., 2015; Scalzo et al., 2015). In contrast, significant correlation was not observed between fruit size and most of the metabolites including anthocyanins in hexaploid and in the diploid accessions, this negative correlation was observed for one year only. However, it is important to note that the different results across the ploidy groups are likely due to the low degree of variation in fruit size and total anthocyanin observed within these accessions, and most importantly the small set of accessions evaluated for these ploidy groups (diploid *N* = 6, hexaploid *N* = 22) (Figure 1 and Supplementary Table S2). Overall, we propose that the results highlighted in the tetraploid accessions are robust and confirm that fruit size is negatively correlated with the metabolites evaluated in this study. Zifkin et al. (2012) localized accumulation anthocyanin and the expression of its biosynthetic genes, specifically in the skin of fully ripen fruits. In another study by Scalzo et al. (2015), anthocyanins are primarily found in the skin of blueberry, so if anthocyanins are extracted from whole berries, small fruit are expected to have relatively higher skin surface area, and would results in higher concentration of metabolites. This would also assume a uniform skin thickness. The fact that in our study fruit surface area, volume and weight are highly correlated, confirms this hypothesis. It is important to note that in blueberry, anthocyanin accumulation in the pulp can occur, but this trait is specific to a small number of accessions or cultivars (Kalt et al., 1999; Ribera et al., 2010) that are not represented in our study. The negative correlation between fruit size and the bioactive metabolites evaluated here, implies that to perform GWAS or QTL mapping studies targeting these bioactive metabolites, estimating fruit size is important since it should be used as a co-factor in the analysis.

To examine the extent of size-independent phenotypic variation for total anthocyanin concentration, the anthocyanin concentration of the tetraploid accessions were normalized by fruit size. The size-independent data showed more than fivefold variation for total anthocyanin (Supplementary Figure S15), indicating the presence of size-independent factors that regulate anthocyanin concentration. The size independent phenotypic variation can be explored to identify other factors such as peel thickness or genes related to the biosynthetic pathway that control this trait. Also, as demonstrated by biplot analysis (Supplementary Figure S4), cultivated accessions with high anthocyanin content and large fruit could be identified. Positive associations were also observed between total anthocyanin with phenolic acid and other flavonoids such as total flavanols and total flavonols (Figures 3, 4). These results suggest that metabolite content and fruit size can be improved simultaneously in breeding programs.

Overall, the results of our study highlighted that the tetraploid accessions evaluated here will be suitable to perform a genome wide association study to investigate the genetic basis controlling flavonoids and polyphenol accumulation for most of the fruit quality traits in this study. Fruit size can be estimated as a proxy of fruit weight or volume and vice versa, and it is a critical parameter to account for when performing genetic studies for the bioactive metabolites. Finally, since the blueberry accessions evaluated in this study represent a publicly available germplasm resource, and the phenotypic data will be made available through the GRIN-Global database, breeders and scientists will be able to use the results of this work as a basis for future genetic studies and in breeding programs.

## Author’s Note

The content of this publication is solely the responsibility of the authors and does not necessarily represent the official views of FFAR.

## Data Availability Statement

The raw data supporting the conclusions of this article will be made available by the authors, without undue reservation, to any qualified researcher.

## Author Contributions

MI designed the study. NB and KH coordinated the fruit harvest and germplasm management. ML coordinated the HPLC and LC-MS analysis. MG and JX performed the HPLC and MS analysis. MM performed the fruit quality phenotyping and all statistical analysis; MM and MI interpreted the results, drafted the sections of the manuscript, prepared the figures and tables, and prepared the final version of the manuscript. MG, JX, CK, NB, KH, MF, and ML critically revised the manuscript. All authors read, reviewed, and approved the manuscript.

## Conflict of Interest

The authors declare that the research was conducted in the absence of any commercial or financial relationships that could be construed as a potential conflict of interest.

## References

[B1] Ballington J. R. (2009). The role of interspecific hybridization in blueberry improvement. *Acta Hortic.* 810 49–60. 10.17660/ActaHortic.2009.810.2

[B2] Bian Y. Ballington J. Raja A. Brouwer C. Reid R. Burke M. (2014). Patterns of simple sequence repeats in cultivated blueberries (*Vaccinium* section *Cyanococcus* spp.) and their use in revealing genetic diversity and population structure. *Mol. Breed.* 34 675–689. 10.1007/s11032-014-0066-7

[B3] Bland J. M. Altman D. (1986). Statistical methods for assessing agreement between two methods of clinical measurement. *Lancet* 327 307–310. 10.1016/s0140-6736(86)90837-8 2868172

[B4] Bourke P. M. Hackett C. A. Voorrips R. E. Visser R. G. F. Maliepaard C. (2019). Quantifying the power and precision of QTL analysis in autopolyploids under bivalent and multivalent genetic models. *G3* 9 2107–2122. 10.1534/g3.119.400269 31036677 PMC6643892

[B5] Campa A. Ferreira J. J. (2018). Genetic diversity assessed by genotyping by sequencing (GBS) and for phenological traits in blueberry cultivars. *PLoS One* 13:e0206361. 10.1371/journal.pone.0206361 30352107 PMC6198992

[B6] Charron C. S. Kurilich A. C. Clevidence B. A. Simon P. W. Harrison D. J. Britz S. J. (2009). Bioavailability of anthocyanins from purple carrot juice: effects of acylation and plant matrix. *J. Agric. Food Chem.* 57 1226–1230. 10.1021/jf802988s 19166298

[B7] Chen W. Gong L. Guo Z. Wang W. Zhang H. Liu X. (2013). A novel integrated method for large-scale detection, identification, and quantification of widely targeted metabolites: application in the study of rice metabolomics. *Mol. Plant* 6 1769–1780. 10.1093/mp/sst080 23702596

[B8] Cheng J. Wei G. Zhou H. Gu C. Vimolmangkang S. Liao L. (2014). Unraveling the mechanism underlying the glycosylation and methylation of anthocyanins in peach. *Plant Physiol.* 166 1044–1058. 10.1104/pp.114.246876 25106821 PMC4213075

[B9] Chong J. Soufan O. Li C. Caraus I. Li S. Bourque G. (2018). MetaboAnalyst 4.0: towards more transparent and integrative metabolomics analysis. *Nucleic Acids Res.* 46 W486–W494. 10.1093/nar/gky310 29762782 PMC6030889

[B10] Colle M. Leisner C. P. Wai C. M. Ou S. Bird K. A. Wang J. (2019). Haplotype-phased genome and evolution of phytonutrient pathways of tetraploid blueberry. *Gigascience* 8:giz012. 10.1093/gigascience/giz012 30715294 PMC6423372

[B11] Correa-Betanzo J. Allen-Vercoe E. McDonald J. Schroeter K. Corredig M. Paliyath G. (2014). Stability and biological activity of wild blueberry (*Vaccinium angustifolium*) polyphenols during simulated in vitro gastrointestinal digestion. *Food Chem.* 165 522–531. 10.1016/j.foodchem.2014.05.135 25038707

[B12] D’Auria J. C. (2006). Acyltransferases in plants: a good time to be BAHD. *Curr. Opin. Plant Biol.* 9 331–340. 10.1016/j.pbi.2006.03.016 16616872

[B13] Diaz-Garcia L. Covarrubias-Pazaran G. Schlautman B. Zalapa J. (2016). GiNA, an efficient and high-throughput software for horticultural phenotyping. *PLoS One* 11:e0160439. 10.1371/journal.pone.0160439 27529547 PMC4986961

[B14] Doligez A. Bertrand Y. Farnos M. Grolier M. Romieu C. Esnault F. (2013). New stable QTLs for berry weight do not colocalize with QTLs for seed traits in cultivated grapevine (*Vitis vinifera* L.). *BMC Plant Biol.* 13:217. 10.1186/1471-2229-13-217 24350702 PMC3878267

[B15] Fleschhut J. Kratzer F. Rechkemmer G. Kulling S. E. (2006). Stability and biotransformation of various dietary anthocyanins in vitro. *Eur. J. Nutr.* 45 7–18. 10.1007/s00394-005-0557-8 15834757

[B16] Georgi L. Johnson-Cicalese J. Honig J. Das S. P. Rajah V. D. Bhattacharya D. (2013). The first genetic map of the American cranberry: exploration of synteny conservation and quantitative trait loci. *Theor. Appl. Genet.* 126 673–692. 10.1007/s00122-012-2010-8 23224333

[B17] Giovanelli G. Buratti S. (2009). Comparison of polyphenolic composition and antioxidant activity of wild Italian blueberries and some cultivated varieties. *Food Chem.* 112 903–908. 10.1016/j.foodchem.2008.06.066

[B18] Giusti M. M. Wrolstad R. E. (2003). Acylated anthocyanins from edible sources and their applications in food systems. *Biochem. Eng. J.* 14 217–225. 10.1016/S1369-703X(02)00221-8

[B19] Grace M. H. Xiong J. Esposito D. Ehlenfeldt M. Lila M. A. (2019). Simultaneous LC-MS quantification of anthocyanins and non-anthocyanin phenolics from blueberries with widely divergent profiles and biological activities. *Food Chem.* 277 336–346. 10.1016/j.foodchem.2018.10.101 30502155 PMC6287264

[B20] Gündüz K. Serçe S. Hancock J. F. (2015). Variation among highbush and rabbiteye cultivars of blueberry for fruit quality and phytochemical characteristics. *J. Food Composit. Anal.* 38 69–79. 10.1016/j.jfca.2014.09.007

[B21] Hancock J. F. Lyrene P. Finn C. E. Vorsa N. Lobos G. A. (2008). Blueberries and cranberries. *Temp. Fruit Crop Breed. Germplasm Genom.* 290 115–149. 10.1007/978-1-4020-6907-9-4

[B22] Hancock J. F. Olmstead J. W. Itle R. A. Callow P. W. Neils-Kraft S. Wheeler E. J. (2018). Performance of an elite, hybrid family of a northern x southern highbush cross (‘Draper’ x ‘Jewel’). *Euphytica* 214:95.

[B23] Jaakola L. (2013). New insights into the regulation of anthocyanin biosynthesis in fruits. *Trends Plant Sci.* 18 477–483. 10.1016/j.tplants.2013.06.003 23870661

[B24] Jaakola L. Määttä K. Pirttilä A. M. Törrönen R. Kärenlampi S. Hohtola A. (2002). Expression of genes involved in anthocyanin biosynthesis in relation to Anthocyanin, Proanthocyanidin, and Flavonol levels during bilberry fruit development. *Plant Physiol.* 130 729–739. 10.1104/pp.006957 12376640 PMC166602

[B25] Kalt W. McDonald J. E. Ricker R. D. Lu X. (1999). Anthocyanin content and profile within and among blueberry species. *Can. J. Plant Sci.* 79 617–623. 10.4141/p99-009

[B26] Kalt W. Ryan D. A. J. Duy J. C. Prior R. L. Ehlenfeldt M. K. Vander Kloet S. P. (2001). Interspecific variation in anthocyanins, phenolics, and antioxidant capacity among genotypes of highbush and lowbush blueberries (*Vaccinium* section *Cyanococcus* spp.). *J. Agric. Food Chem.* 49 4761–4767. 10.1021/jf010653e 11600018

[B27] Krikorian R. Shidler M. D. Nash T. A. Kalt W. Vinqvist-Tymchuk M. R. Shukitt-Hale B. (2010). Blueberry supplementation improves memory in older adults. *J. Agric. Food Chem.* 58 3996–4000. 10.1021/jf9029332 20047325 PMC2850944

[B28] Kurilich A. C. Clevidence B. A. Britz S. J. Simon P. W. Novotny J. A. (2005). Plasma and urine responses are lower for acylated vs nonacylated anthocyanins from raw and cooked purple carrots. *J. Agric. Food Chem.* 53 6537–6542. 10.1021/jf050570o 16076146

[B29] Lê S. Josse J. Husson F. (2008). FactoMineR: an R package for multivariate analysis. *J. Statist. Softw.* 25 1–18.

[B30] Le Roy J. Huss B. Creach A. Hawkins S. Neutelings G. (2016). Glycosylation is a major regulator of phenylpropanoid availability and biological activity in plants. *Front. Plant Sci.* 7:735. 10.3389/fpls.2016.00735 27303427 PMC4880792

[B31] Li D. Li B. Ma Y. Sun X. Lin Y. Meng X. (2017). Polyphenols, anthocyanins, and flavonoids contents and the antioxidant capacity of various cultivars of highbush and half-high blueberries. *J. Food Composit. Anal.* 62 84–93. 10.1016/j.jfca.2017.03.006

[B32] Li L. Zhang H. Liu Z. Cui X. Zhang T. Li Y. (2016). Comparative transcriptome sequencing and de novo analysis of *Vaccinium corymbosum* during fruit and color development. *BMC Plant Biol.* 16:223. 10.1186/s12870-016-0866-5 27729032 PMC5059916

[B33] Lin Y. Wang Y. Li B. Tan H. Li D. Li L. (2018). Comparative transcriptome analysis of genes involved in anthocyanin synthesis in blueberry. *Plant Physiol. Biochem.* 127 561–572. 10.1016/j.plaphy.2018.04.034 29727860

[B34] Lobos G. A. Hancock J. F. (2015). Breeding blueberries for a changing global environment: a review. *Front. Plant Sci.* 6:782. 10.3389/fpls.2015.00782 26483803 PMC4588112

[B35] Lourenço V. M. Rodrigues P. C. Pires A. M. Piepho H.-P. (2017). A robust DF-REML framework for variance components estimation in genetic studies. *Bioinformatics* 33 3584–3594. 10.1093/bioinformatics/btx457 29036274

[B36] Lyrene P. M. Vorsa N. Ballington J. R. (2003). Polyploidy and sexual polyploidization in the genus *Vaccinium*. *Euphytica* 133 27–36. 10.1023/A:1025608408727

[B37] Martineau L. C. Couture A. Spoor D. Benhaddou-Andaloussi A. Harris C. Meddah B. (2006). Anti-diabetic properties of the Canadian Lowbush blueberry *Vaccinium angustifolium* Ait. *Phytomedicine* 13 612–623. 10.1016/j.phymed.2006.08.005 16979328

[B38] Matera R. Gabbanini S. Berretti S. Amorati R. De Nicola G. R. Iori R. (2015). Acylated anthocyanins from sprouts of *Raphanus sativus* cv. sango: isolation, structure elucidation and antioxidant activity. *Food Chem.* 166 397–406. 10.1016/j.foodchem.2014.06.056 25053073

[B39] Matsuda F. Nakabayashi R. Yang Z. Okazaki Y. Yonemaru J. Ebana K. (2015). Metabolome-genome-wide association study dissects genetic architecture for generating natural variation in rice secondary metabolism. *Plant J.* 81 13–23. 10.1111/tpj.12681 25267402 PMC4309412

[B40] McDougall G. J. Fyffe S. Dobson P. Stewart D. (2007). Anthocyanins from red cabbage–stability to simulated gastrointestinal digestion. *Phytochemistry* 68 1285–1294. 10.1016/j.phytochem.2007.02.004 17382979

[B41] Mengist M. F. Alves S. Griffin D. Creedon J. McLaughlin M. J. Jones P. W. (2018). Genetic mapping of quantitative trait loci for tuber-cadmium and zinc concentration in potato reveals associations with maturity and both overlapping and independent components of genetic control. *Theor. Appl. Genet.* 131 929–945. 10.1007/s00122-017-3048-4 29307117

[B42] Noguchi A. Horikawa M. Fukui Y. Fukuchi-Mizutani M. Iuchi-Okada A. Ishiguro M. (2009). Local differentiation of sugar donor specificity of flavonoid glycosyltransferase in lamiales W. *Plant Cell* 21 1556–1572. 10.1105/tpc.108.063826 19454730 PMC2700533

[B43] Norberto S. Silva S. Meireles M. Faria A. Pintado M. Calhau C. (2013). Blueberry anthocyanins in health promotion: a metabolic overview. *J. Funct. Foods* 5 1518–1528. 10.1016/j.jff.2013.08.015

[B44] Oliveira H. Perez-Gregório R. de Freitas V. Mateus N. Fernandes I. (2019). Comparison of the in vitro gastrointestinal bioavailability of acylated and non-acylated anthocyanins: purple-fleshed sweet potato vs red wine. *Food Chem.* 276 410–418. 10.1016/j.foodchem.2018.09.159 30409613

[B45] Prencipe F. P. Bruni R. Guerrini A. Rossi D. Benvenuti S. Pellati F. (2014). Metabolite profiling of polyphenols in *Vaccinium berries* and determination of their chemopreventive properties. *J. Pharmaceut. Biomed. Anal.* 89 257–267. 10.1016/j.jpba.2013.11.016 24316426

[B46] Retamales J. B. Hancock J. F. (2018). *Blueberries.* Wallington: CABI.

[B47] Ribera A. E. Reyes-Diaz M. Alberdi M. Zuñiga G. E. Mora M. L. (2010). Antioxidant compounds in skin and pulp of fruits change among genotypes and maturity stages in highbush blueberry (*Vaccinium corymbosum* L.) grown in southern Chile. *J. Soil Sci. Plant Nutr.* 10 509–536. 10.4067/s0718-95162010000200010

[B48] Rodriguez-Mateos A. Cifuentes-Gomez T. Tabatabaee S. Lecras C. Spencer J. P. E. (2012). Procyanidin, anthocyanin, and chlorogenic acid contents of highbush and lowbush blueberries. *J. Agric. Food Chem.* 60 5772–5778. 10.1021/jf203812w 22175691

[B49] Rodriguez-Saona C. Vincent C. Isaacs R. (2019). Blueberry IPM: past successes and future challenges. *Ann. Rev. Entomol.* 64 95–114. 10.1146/annurev-ento-011118-112147 30629894

[B50] Scalzo J. Stevenson D. Hedderley D. (2013). Blueberry estimated harvest from seven new cultivars: fruit and anthocyanins. *Food Chem.* 139 44–50. 10.1016/j.foodchem.2013.01.091 23561076

[B51] Scalzo J. Stevenson D. Hedderley D. (2015). Polyphenol compounds and other quality traits in blueberry cultivars. *J. Berry Res.* 5 117–130. 10.3233/JBR-150097

[B52] Schepker H. Kowarik I. (1998). “Invasive north American blueberry hybrids (vaccinium corymbosum x angustifolium) in northern germany,” in *Plant Invasions: Ecological Mechanims and Human Responses*, eds Starfinger U. Edwards K. Kowarik M. Williamson M. (Leiden: Backhuys Publishers).

[B53] Schlautman B. Covarrubias-Pazaran G. Diaz-Garcia L. A. Johnson-Cicalese J. Iorrizo M. Rodriguez-Bonilla L. (2015). Development of a high-density cranberry SSR linkage map for comparative genetic analysis and trait detection. *Mol. Breed.* 35 1–18. 10.1007/s11032-015-0367-5

[B54] Schmidt P. Hartung J. Bennewitz J. Piepho H.-P. (2019). Heritability in plant breeding on a genotype-difference basis, Genetics. *Genetics* 212 991–1008. 10.1534/genetics.119.302134 31248886 PMC6707473

[B55] Strauch R. C. Mengist M. F. Pan K. Yousef G. G. Iorizzo M. Brown A. F. (2019). Variation in anthocyanin profiles of 27 genotypes of red cabbage over two growing seasons. *Food Chem.* 301:125289. 10.1016/j.foodchem.2019.125289 31387047

[B56] Stull A. J. Cash K. C. Johnson W. D. Champagne C. M. Cefalu W. T. (2010). Bioactives in blueberries improve insulin sensitivity in obese, insulin-resistant men and women. *J. Nutr.* 140 1764–1768. 10.3945/jn.110.125336 20724487 PMC3139238

[B57] Sun Y. Nemec-Bakk A. S. Mallik A. U. Bagchi A. K. Singal P. K. Khaper N. (2019). Blueberry extract attenuates doxorubicin-induced damage in H9c2 cardiac cells. *Can. J. Physiol. Pharmacol.* 97 880–884. 10.1139/cjpp-2019-0031 31365282

[B58] Szymańska E. Saccenti E. Smilde A. K. Westerhuis J. A. (2012). Double-check: validation of diagnostic statistics for PLS-DA models in metabolomics studies. *Metabolomics* 8 3–16. 10.1007/s11306-011-0330-3 22593721 PMC3337399

[B59] Timmers M. A. Grace M. H. Yousef G. G. Lila M. A. (2017). Inter-and intra-seasonal changes in anthocyanin accumulation and global metabolite profiling of six blueberry genotypes. *J. Food Composit. Anal.* 59 105–110. 10.1016/j.jfca.2017.02.019

[B60] Wang H. Wang C. Fan W. Yang J. Appelhagen I. Wu Y. (2018). A novel glycosyltransferase catalyses the transfer of glucose to glucosylated anthocyanins in purple sweet potato. *J. Exp. Bot.* 69 5445–5459. 10.1093/jxb/ery305 30124996 PMC6255700

[B61] Wang Y. Fong S. K. Singh A. P. Vorsa N. Johnson-Cicalese J. (2019). Variation of Anthocyanins, Proanthocyanidins, Flavonols, and organic acids in cultivated and wild diploid blueberry species. *Hortscience* 54 576–585. 10.21273/hortsci13491-18

[B62] Warnes M. G. R. Bolker B. Bonebakker L. Gentleman R. (2016). *Package ‘gplots.’ Various R Programming Tools for Plotting Data.* Available online at: https://github.com/talgalili/gplots (accessed July 28, 2019).

[B63] Wei T. Simko V. Levy M. Xie Y. Jin Y. Zemla J. (2017). Package ‘corrplot.’. *Statistician* 56 316–324.

[B64] Wen W. Li D. Li X. Gao Y. Li W. Li H. (2014). Metabolome-based genome-wide association study of maize kernel leads to novel biochemical insights. *Nat. Commun.* 5:3438. 10.1038/ncomms4438 24633423 PMC3959190

[B65] Yang H. Tian T. Wu D. Guo D. Lu J. (2019). Prevention and treatment effects of edible berries for three deadly diseases: cardiovascular disease, cancer and diabetes. *Crit. Rev. Food Sci. Nutr.* 59 1903–1912. 10.1080/10408398.2018.1432562 29381386

[B66] Yang W. Kortesniemi M. Ma X. Zheng J. Yang B. (2019). Enzymatic acylation of blackcurrant (*Ribes nigrum*) anthocyanins and evaluation of lipophilic properties and antioxidant capacity of derivatives. *Food Chem.* 281 189–196. 10.1016/j.foodchem.2018.12.111 30658747

[B67] Yousef G. G. Brown A. F. Funakoshi Y. Mbeunkui F. Grace M. H. Ballington J. R. (2013). Efficient quantification of the health-relevant anthocyanin and phenolic acid profiles in commercial cultivars and breeding selections of blueberries (*Vaccinium* spp.). *J. Agric. Food Chem.* 61 4806–4815. 10.1021/jf400823s 23635035

[B68] Yousef G. G. Lila M. A. Guzman I. Ballington J. R. Brown A. F. (2014). Impact of interspecific introgression on anthocyanin profiles of southern highbush blueberry. *J. Am. Soc. Hortic. Sci.* 139 99–112. 10.21273/jashs.139.2.99

[B69] Zhao C. L. Yu Y. Q. Chen Z. J. Wen G. S. Wei F. G. Zheng Q. (2017). Stability-increasing effects of anthocyanin glycosyl acylation. *Food Chem.* 214 119–128. 10.1016/j.foodchem.2016.07.073 27507456

[B70] Zifkin M. Jin A. Ozga J. A. Zaharia L. I. Schernthaner J. P. Gesell A. (2012). Gene expression and metabolite profiling of developing highbush blueberry fruit indicates transcriptional regulation of flavonoid metabolism and activation of abscisic acid metabolism. *Plant Physiol.* 158 200–224. 10.1104/pp.111.180950 22086422 PMC3252089

[B71] Zong Y. Kang H. Fang Q. Chen X. Zhou M. Ni J. (2019). Phylogenetic relationship and genetic background of blueberry (*Vaccinium* spp.) based on retrotransposon-based SSAP molecular markers. *Sci. Hortic.* 247 116–122. 10.1016/j.scienta.2018.11.017

[B72] Zoratti L. Jaakola L. Häggman H. Giongo L. (2015). Anthocyanin profile in berries of wild and cultivated *Vaccinium* spp. along altitudinal gradients in the Alps. *J. Agric. Food Chem.* 63 8641–8650. 10.1021/acs.jafc.5b02833 26373665

