# Supplementary material for: Diversity in Metabolites and Fruit Quality Traits in Blueberry Enables Ploidy and Species Differentiation and Establishes a Strategy for Future Genetic Studies
Source: Front Plant Sci. 2020 Apr 3;11:370. doi: 10.3389/fpls.2020.00370 (PMC7147330; doi:10.3389/fpls.2020.00370)
Supplement: Supplementary file 1 [file Data_Sheet_1.PDF]

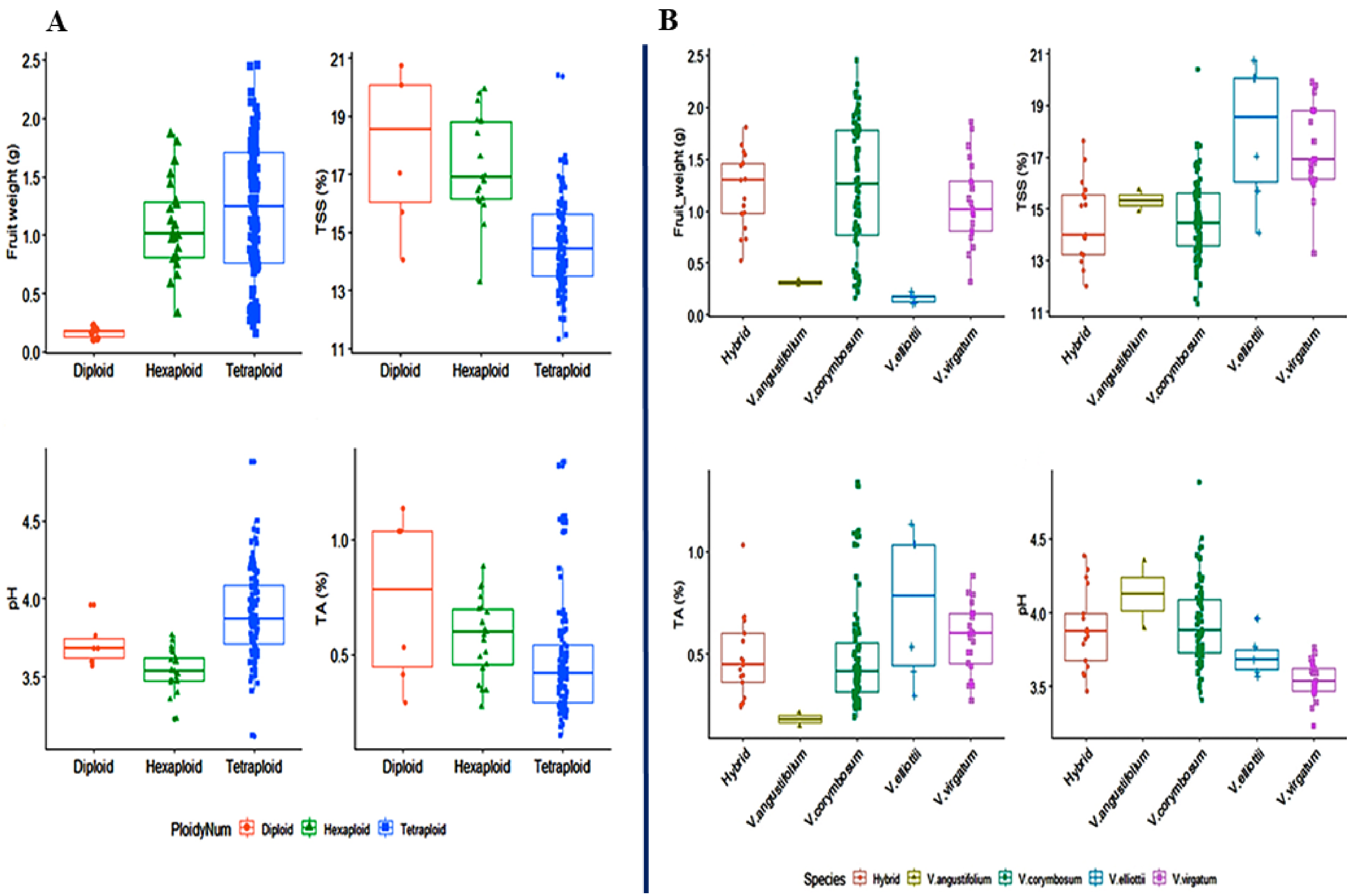

Supplementary Figure S1. Boxplot diagrams depicting the effect of ploidy and species to metabolite and fruit quality traits. (A) Boxplot showing variation within and between ploidy levels; (B) boxplot showing variations among different species.

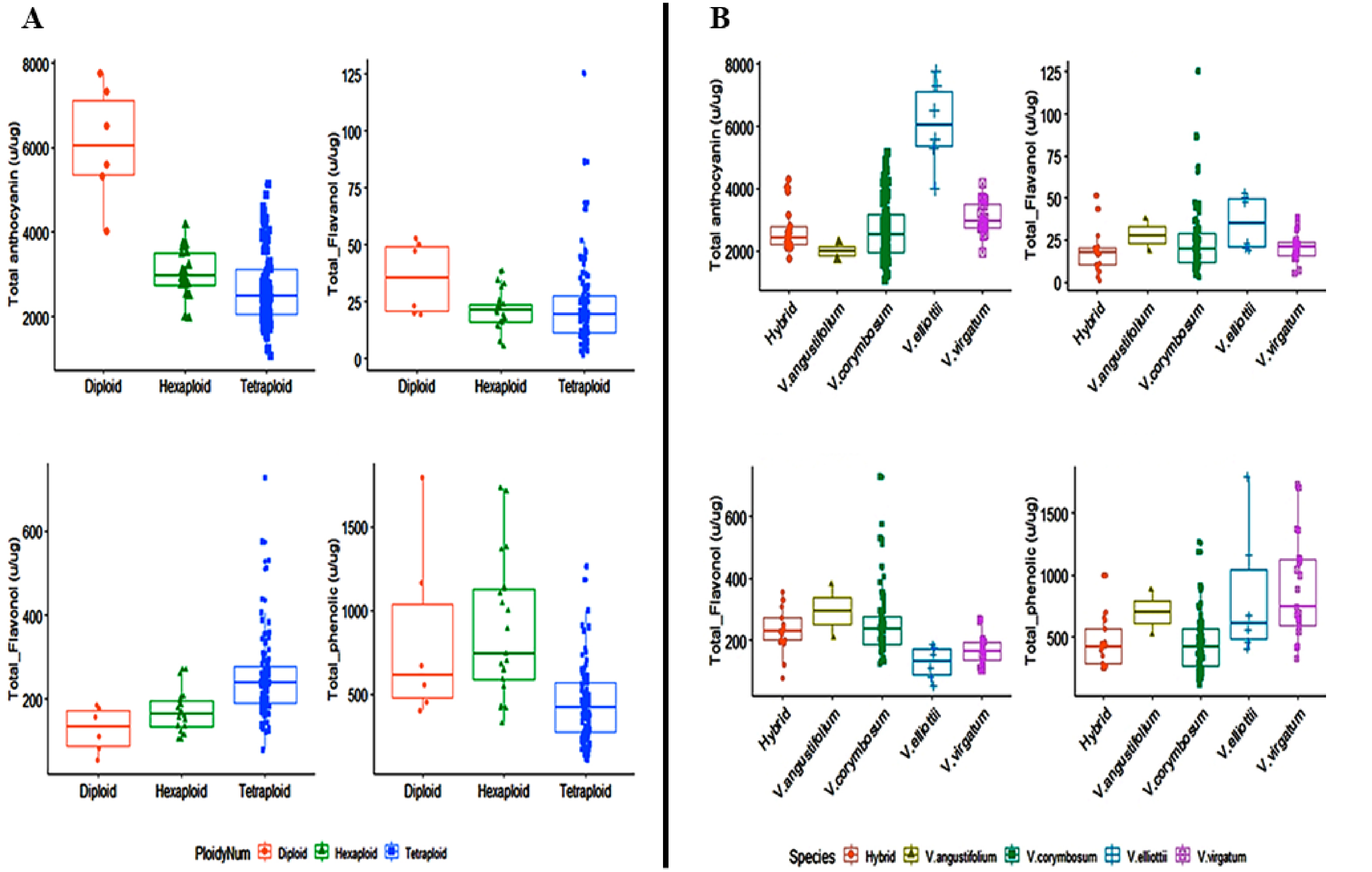

Continue Supplementary Figure S1. Boxplot diagrams depicting the effect of ploidy and species to metabolite and fruit quality traits. (A) Boxplot showing variation within and between ploidy levels; (B) boxplot showing variations among different species.

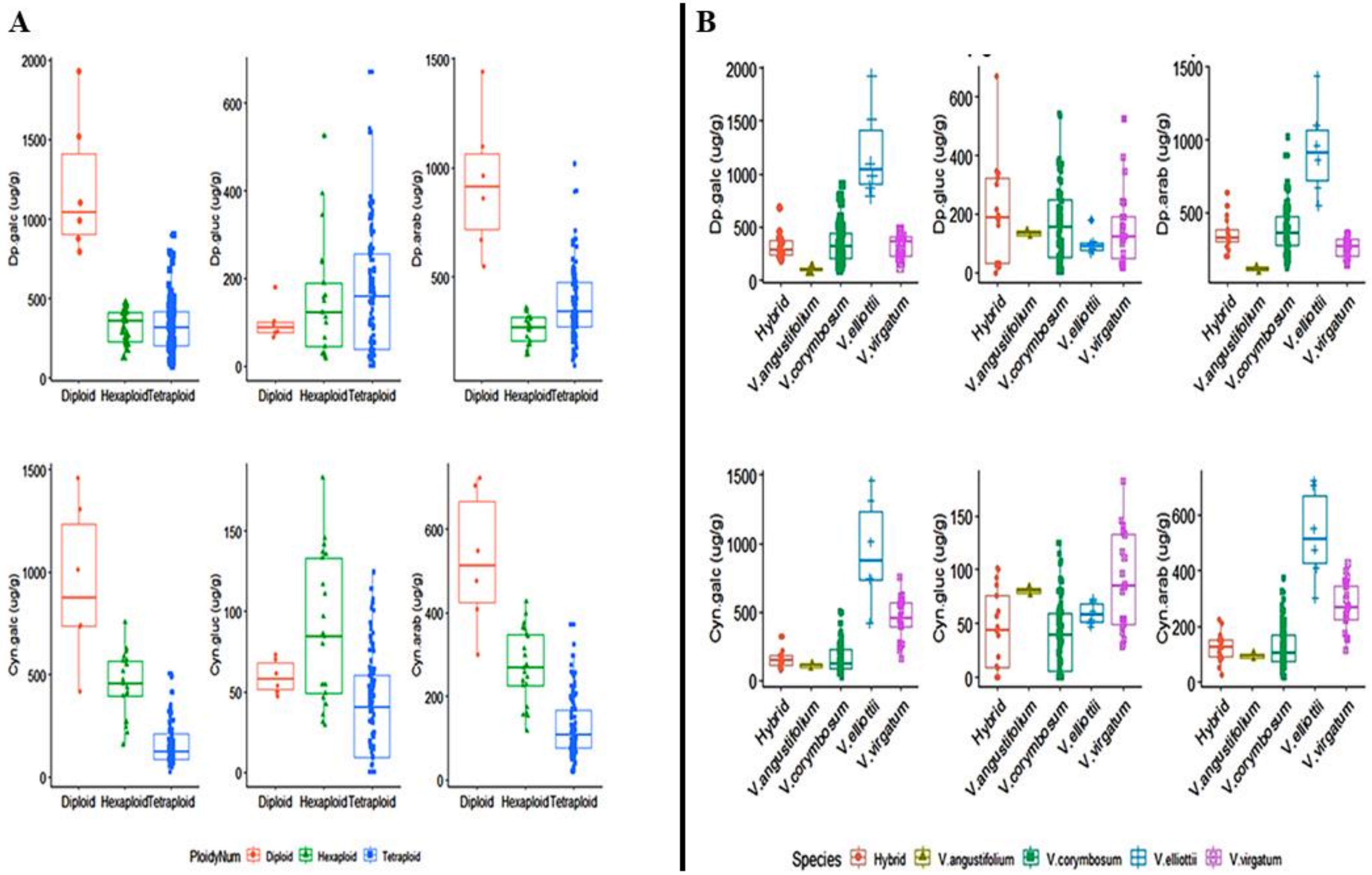

Continue Supplementary Figure S1. Boxplot diagrams depicting the effect of ploidy and species to metabolite and fruit quality traits. (A) Boxplot showing variation within and between ploidy levels; (B) boxplot showing variations among different species.

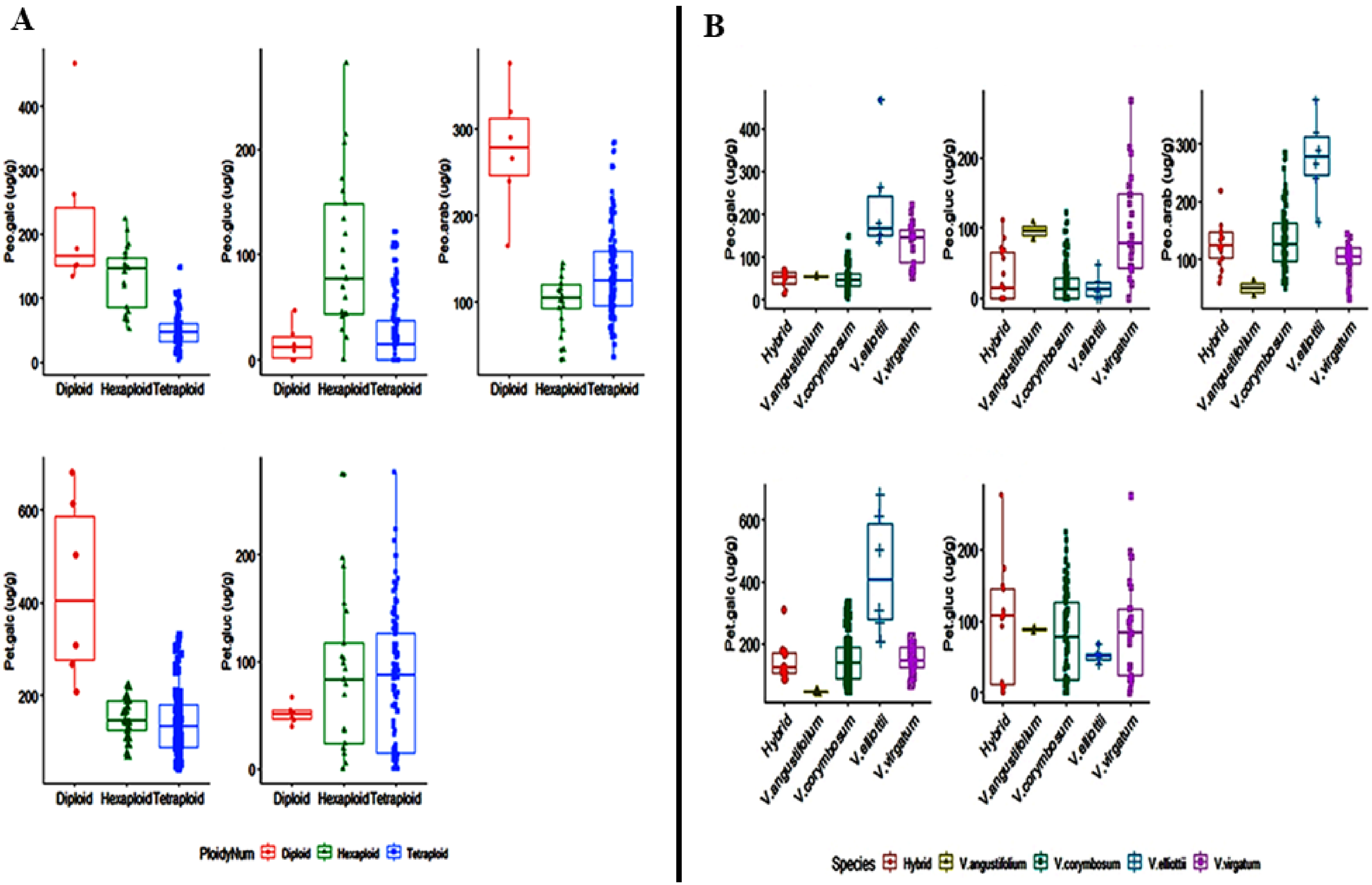

Continue Supplementary Figure S1. Boxplot diagrams depicting the effect of ploidy and species to metabolite and fruit quality traits. (A) Boxplot showing variation within and between ploidy levels; (B) boxplot showing variations among different species.

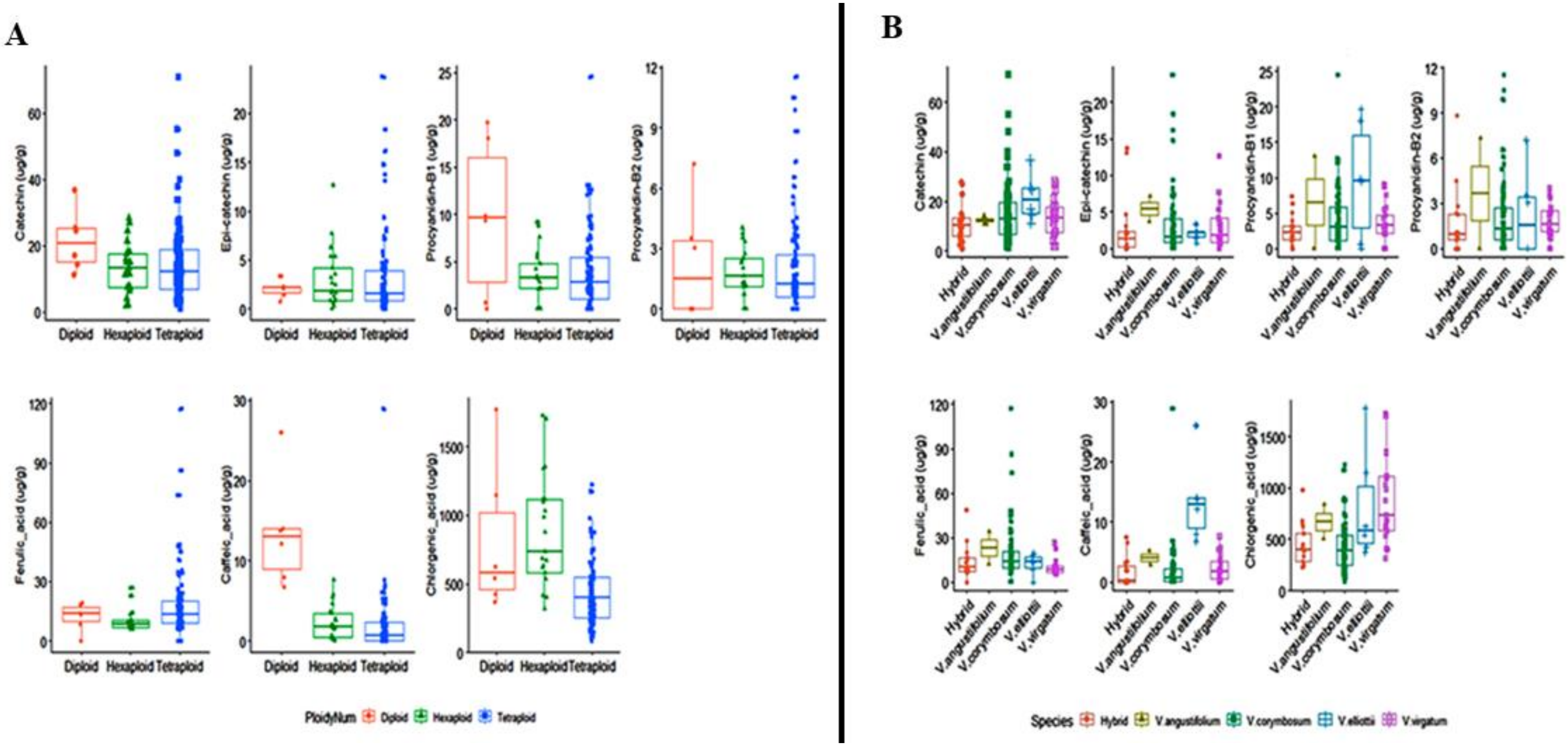

Continue Supplementary Figure S1. Boxplot diagrams depicting the effect of ploidy and species to metabolite and fruit quality traits. (A) Boxplot showing variation within and between ploidy levels; (B) boxplot showing variations among different species.

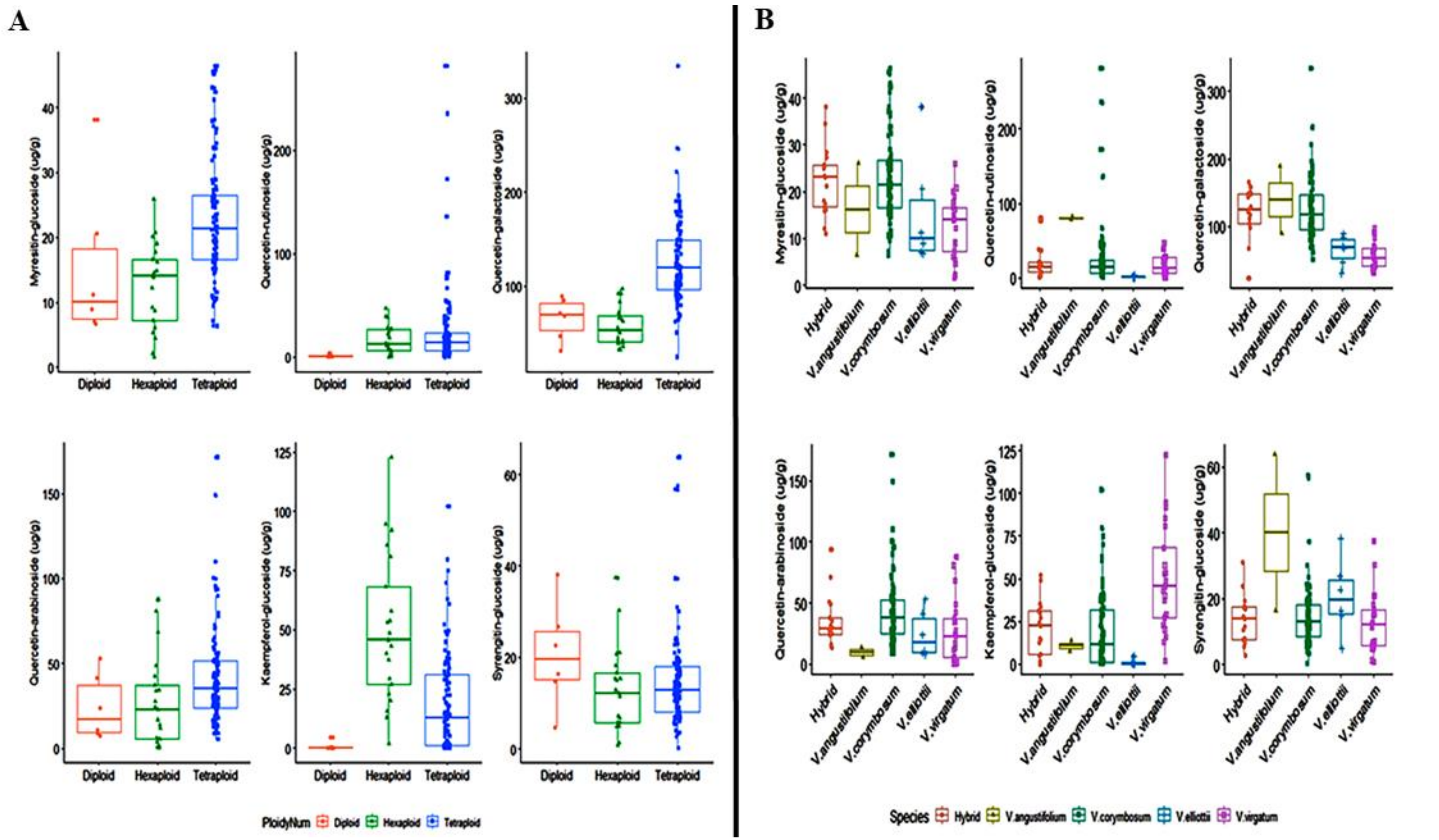

Continue Supplementary Figure S1. Boxplot diagrams depicting the effect of ploidy and species to metabolite and fruit quality traits. (A) Boxplot showing variation within and between ploidy levels; (B) boxplot showing variations among different species.

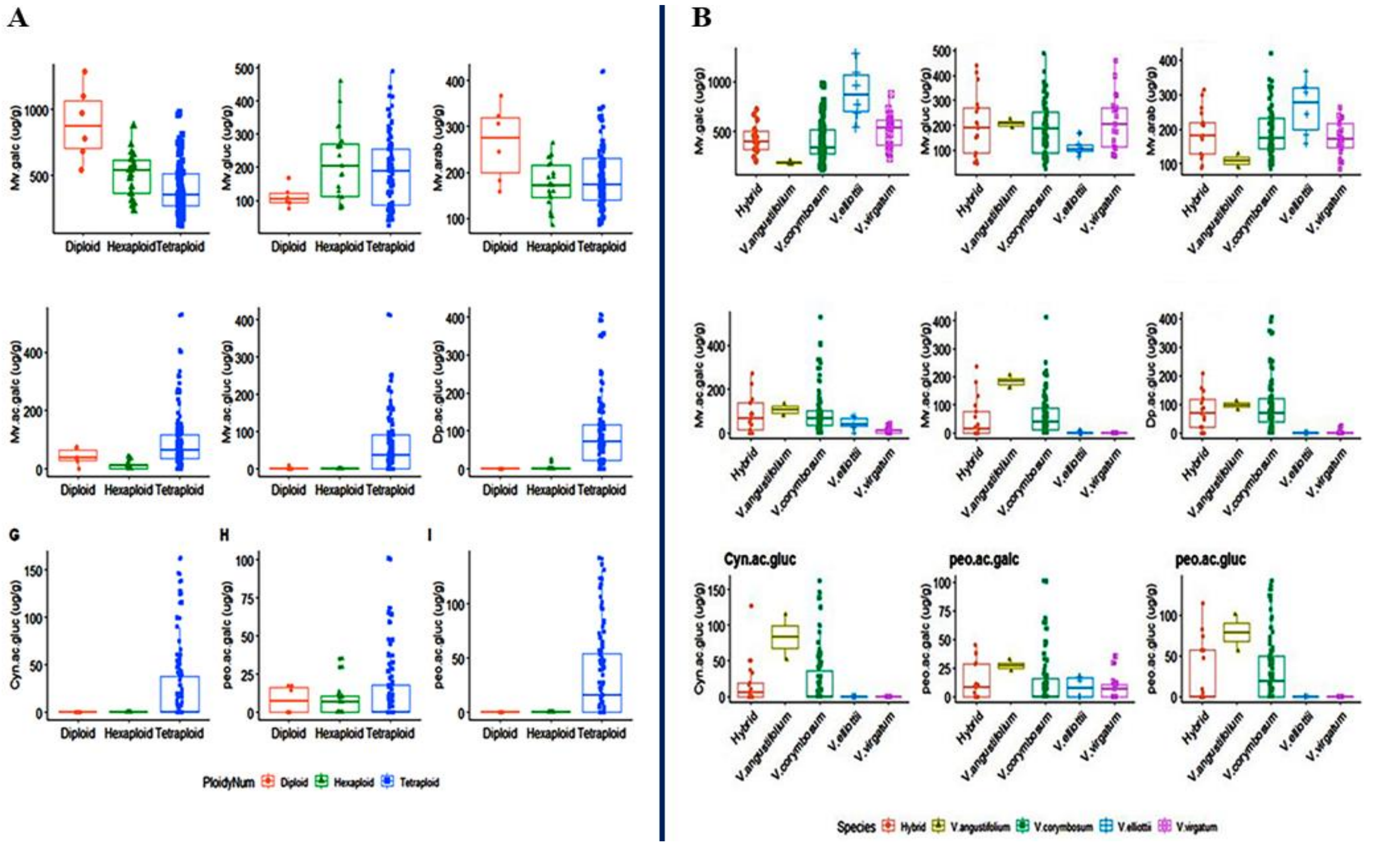

Continue Supplementary Figure S1. Boxplot diagrams depicting the effect of ploidy and species to metabolite and fruit quality traits. (A) Boxplot showing variation within and between ploidy levels; (B) boxplot showing variations among different species.

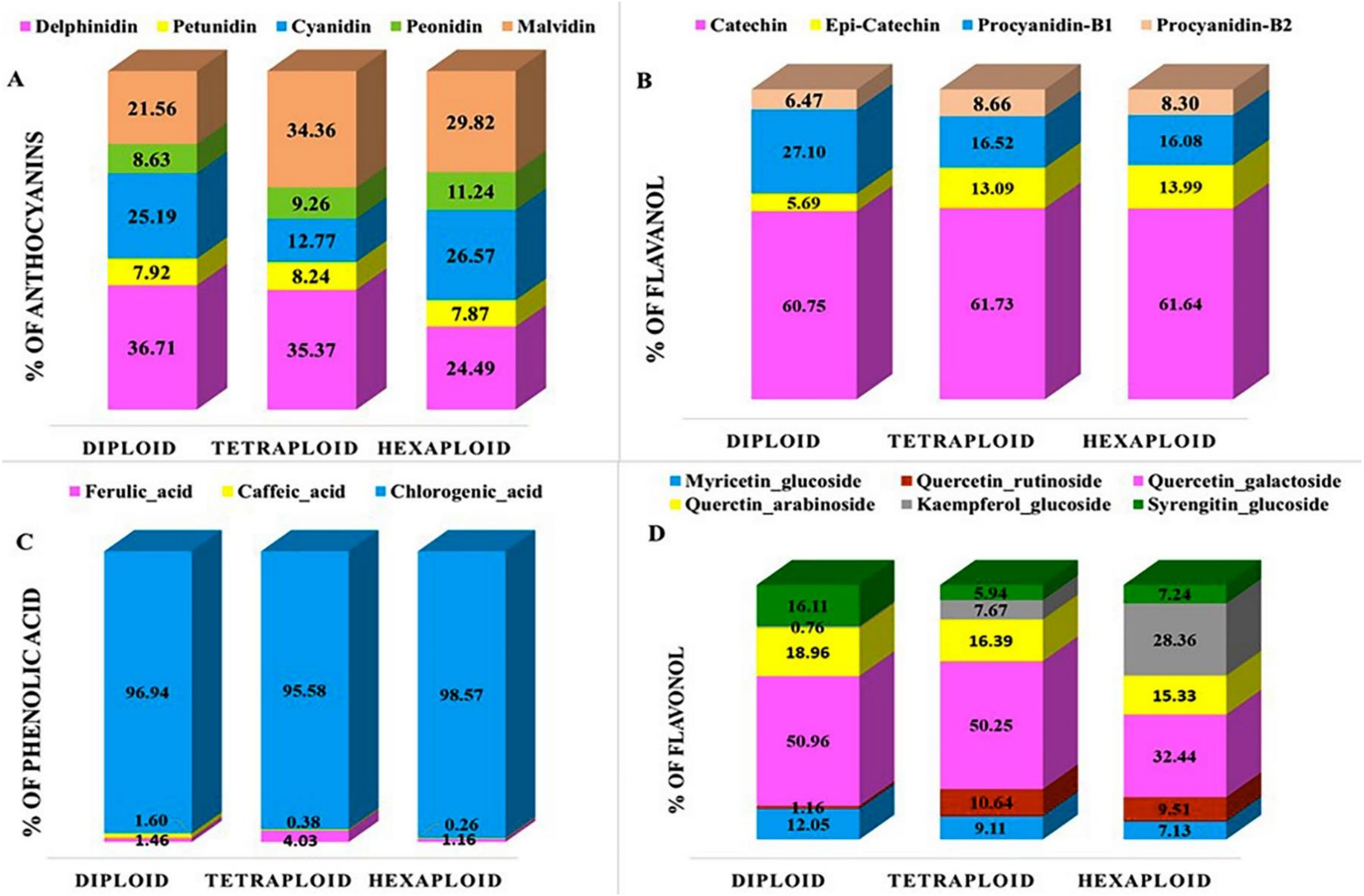

Supplementary Figure S2. Relative content (%) of anthocyanins (A), flavanols (B), phenolic acids (C) and flavonols (D) in diploid, tetraploid and hexaploid accessions. Two-year data from all accessions belonging to each ploidy group (2x, 4x, 6x) were used to calculate the average content and calculate the relative content (%).

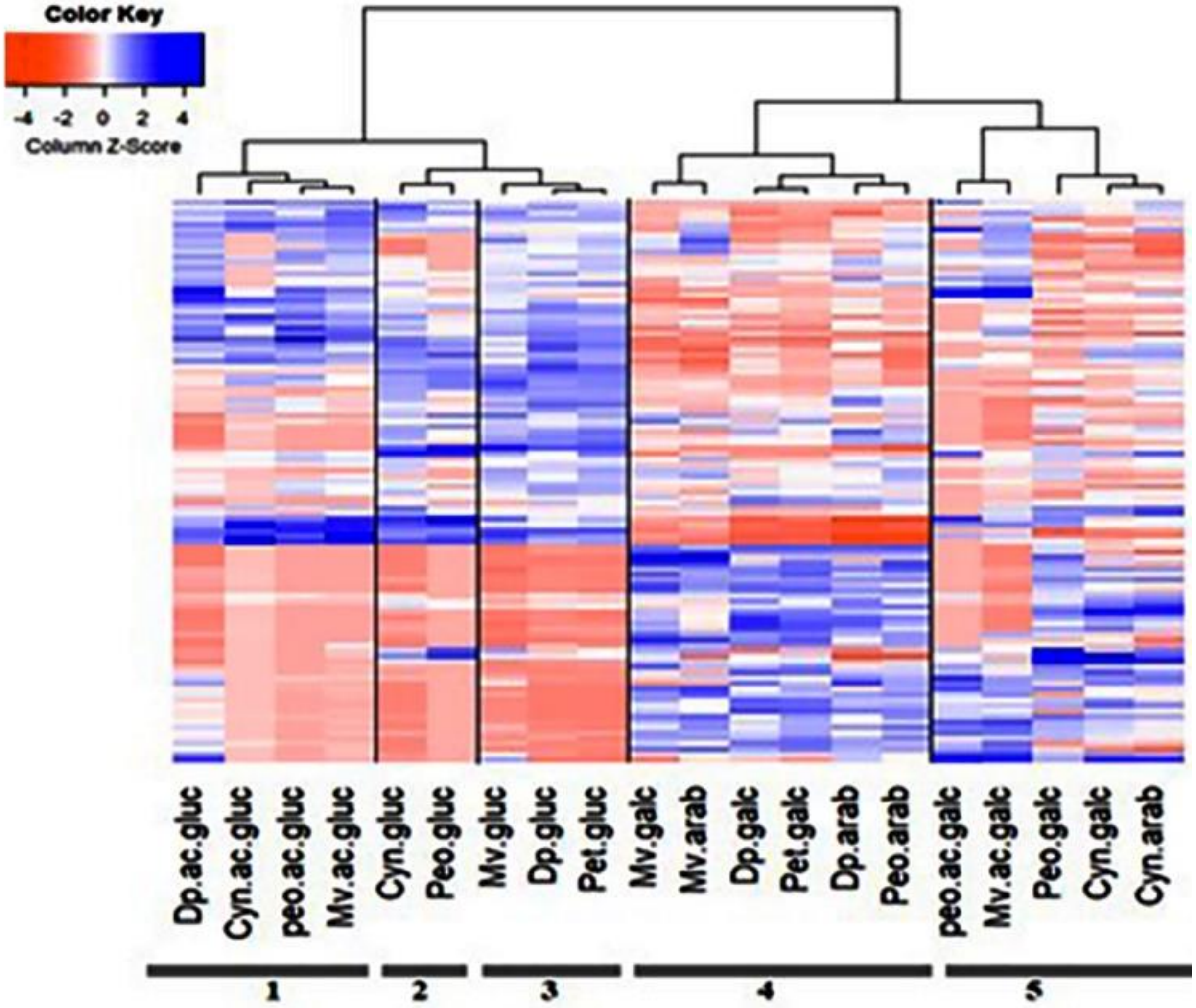

Supplementary Figure S3. Hierarchical clustering analysis of the relative % contribution of individual anthocyanin to the total anthocyanin. Numbers 1 to 5 showing the different clusters of anthocyanins, acylated glucoside containing anthocyanins (1) non-acylated glucoside containing cyanidin derivative anthocyanins; (2), glucoside containing delphinidin derivative anthocyanins; (3), arabinoside/galactoside containing anthocyanins (4, 5).

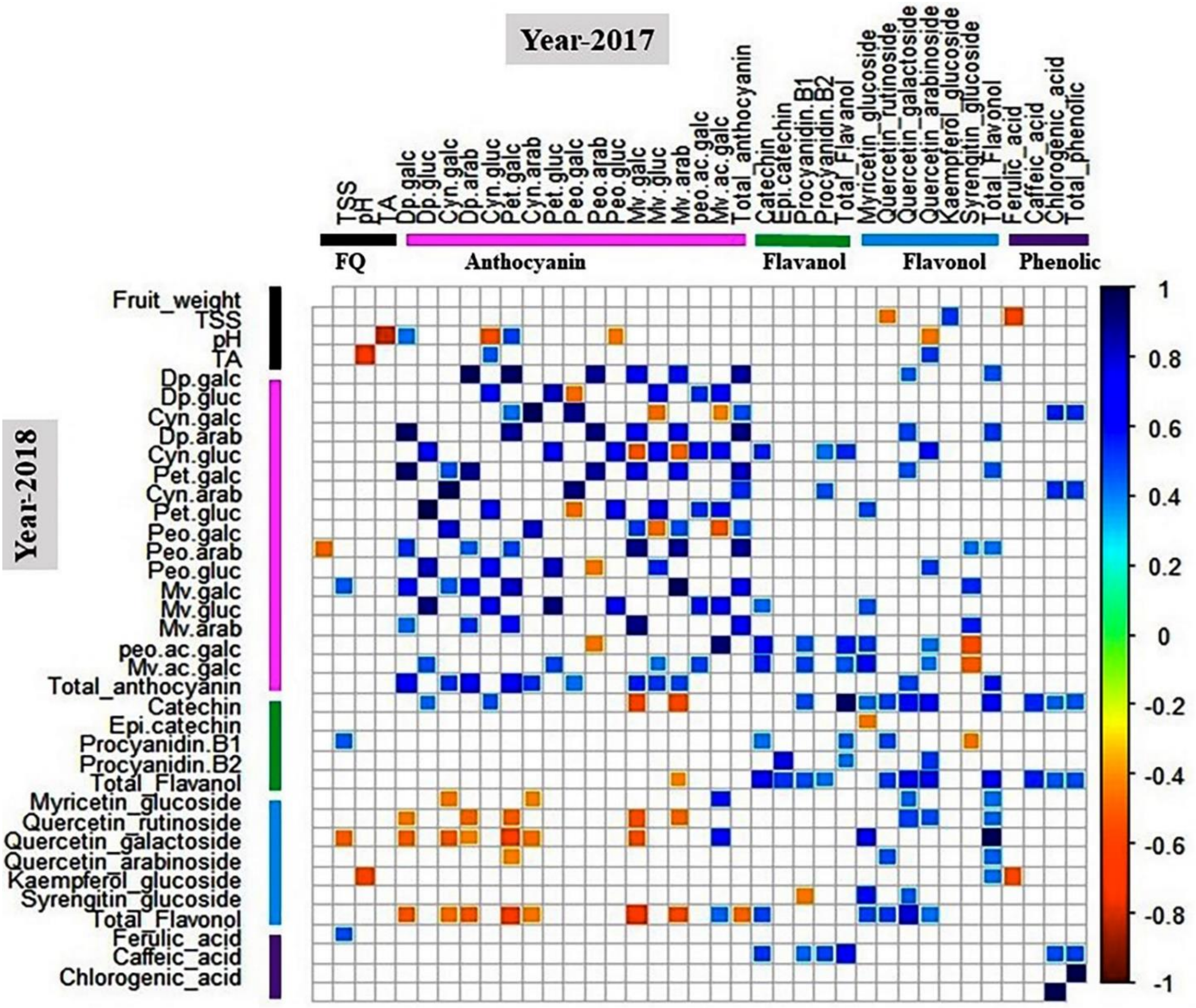

Supplementary Figure S4. Correlation analysis of metabolite and fruit quality traits for 22 hexaploid accessions. White boxes represent non-significant correlations. Color bar indicates metabolite classes and fruit quality traits. Cyan-to-blue and yellow-to-red colors showing significant (*$P < 0.05$*) positive and negative correlation between traits, respectively. Abbreviation: FQ, fruit quality trait.

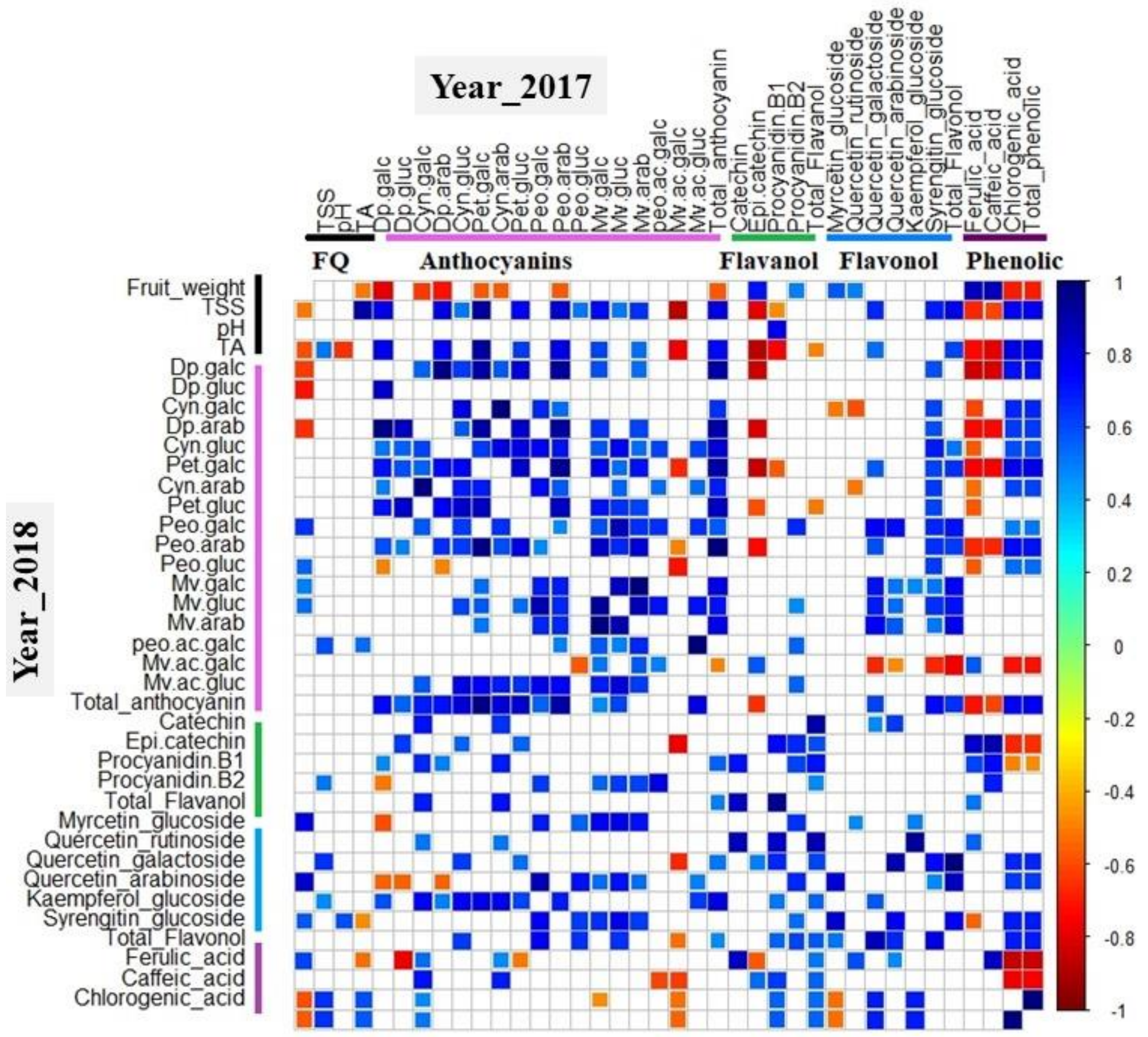

Supplementary Figure S5. Correlation analysis of metabolite and fruit quality traits for 6 diploid accessions. White boxes represent non-significant correlations. Color bar indicates metabolite classes and fruit quality traits. Cyan-to-blue and yellow-to-red colors showing significant (*P < 0.05*) positive and negative correlation between traits, respectively. Abbreviation: FQ, fruit quality trait.

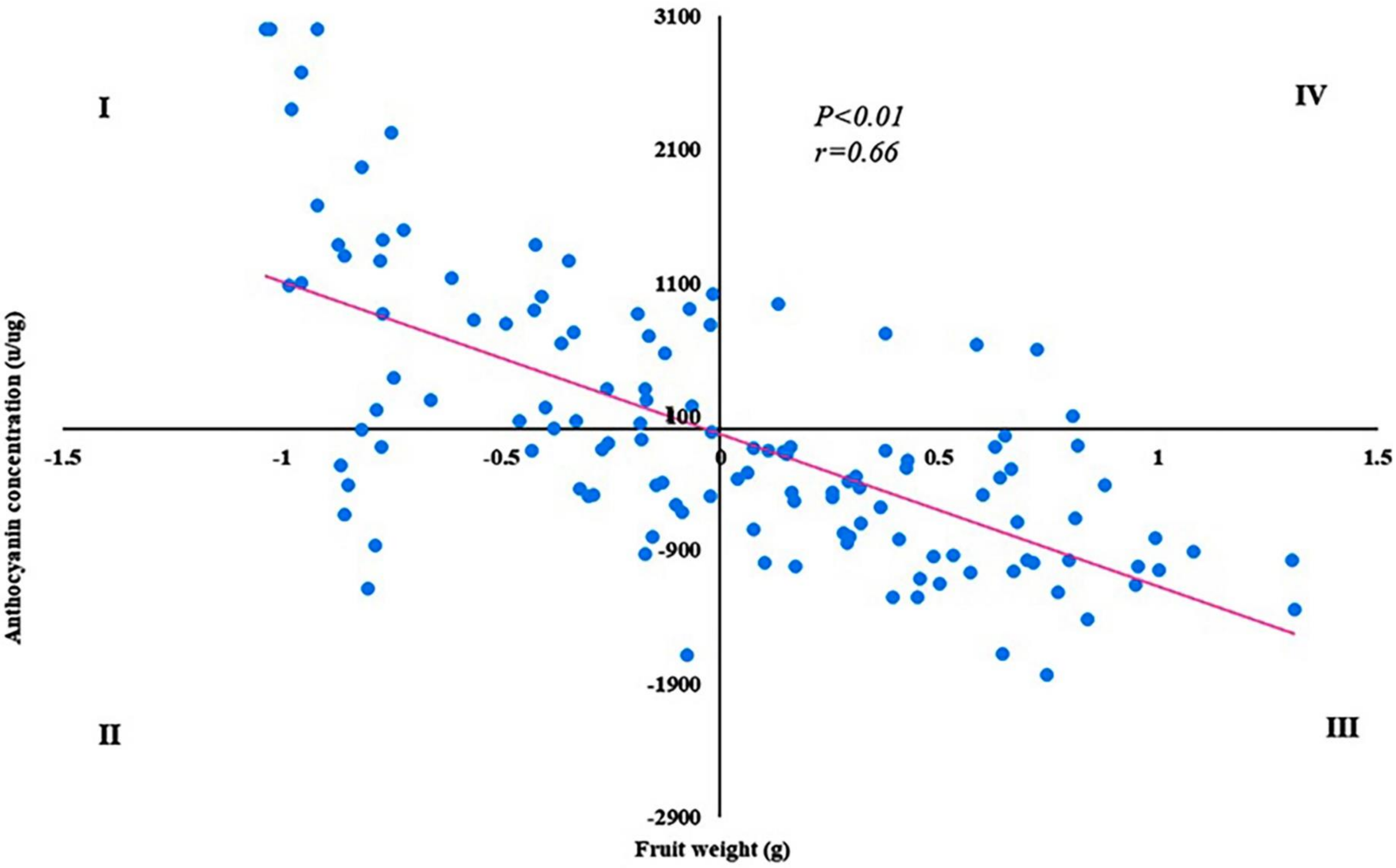

Supplementary Figure 6. Correlation between fruit weight and total anthocyanin content in 100 tetraploid accessions. The total anthocyanin content and fruit weight of each accession was compared with the grand mean, total anthocyanins (2,686 ug/g) and fruit weight (1.24 g). Each of the four quadrants indicate: high (>2686 ug/g) total anthocyanin content and low (< 1.24 g) fruit weight (I), low ((<2686 ug/g) anthocyanin content and low fruit weight (< 1.24 g) (II), low anthocyanin content (< 2686 ug/g) and high (> 1.24 g) fruit weight (III) and high anthocyanin content ((>2686 ug/g) and high fruit weight (> 1.24 g) (IV).

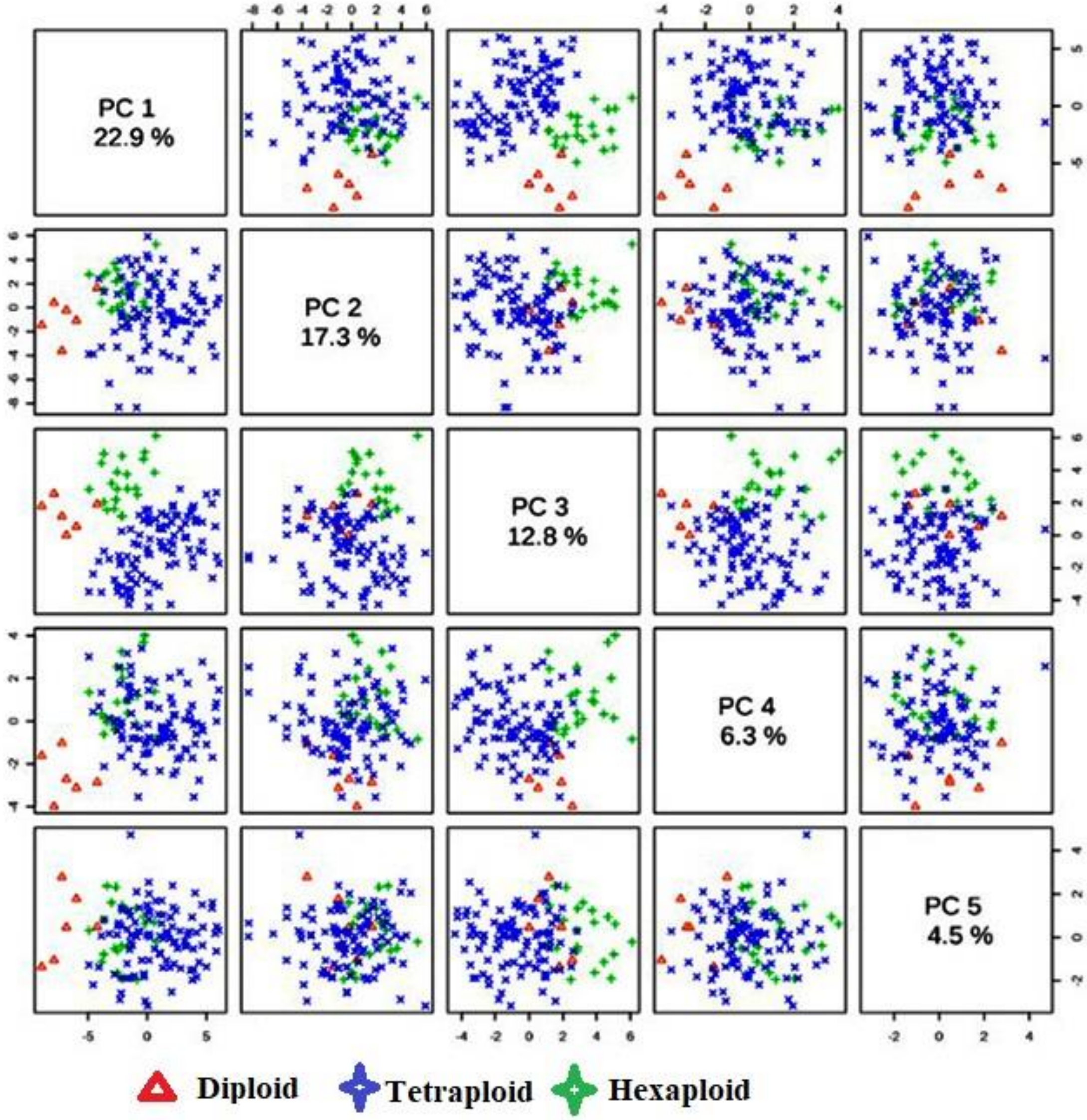

Supplementary figure S7. PCA score plots (PC1-PC5) of metabolite and fruit quality data from the 128 blueberry accessions. The percentage indicated by each component label represents the relative variation explained by each component. Diploid, tetraploid, and hexaploid accessions were identified with different colors.

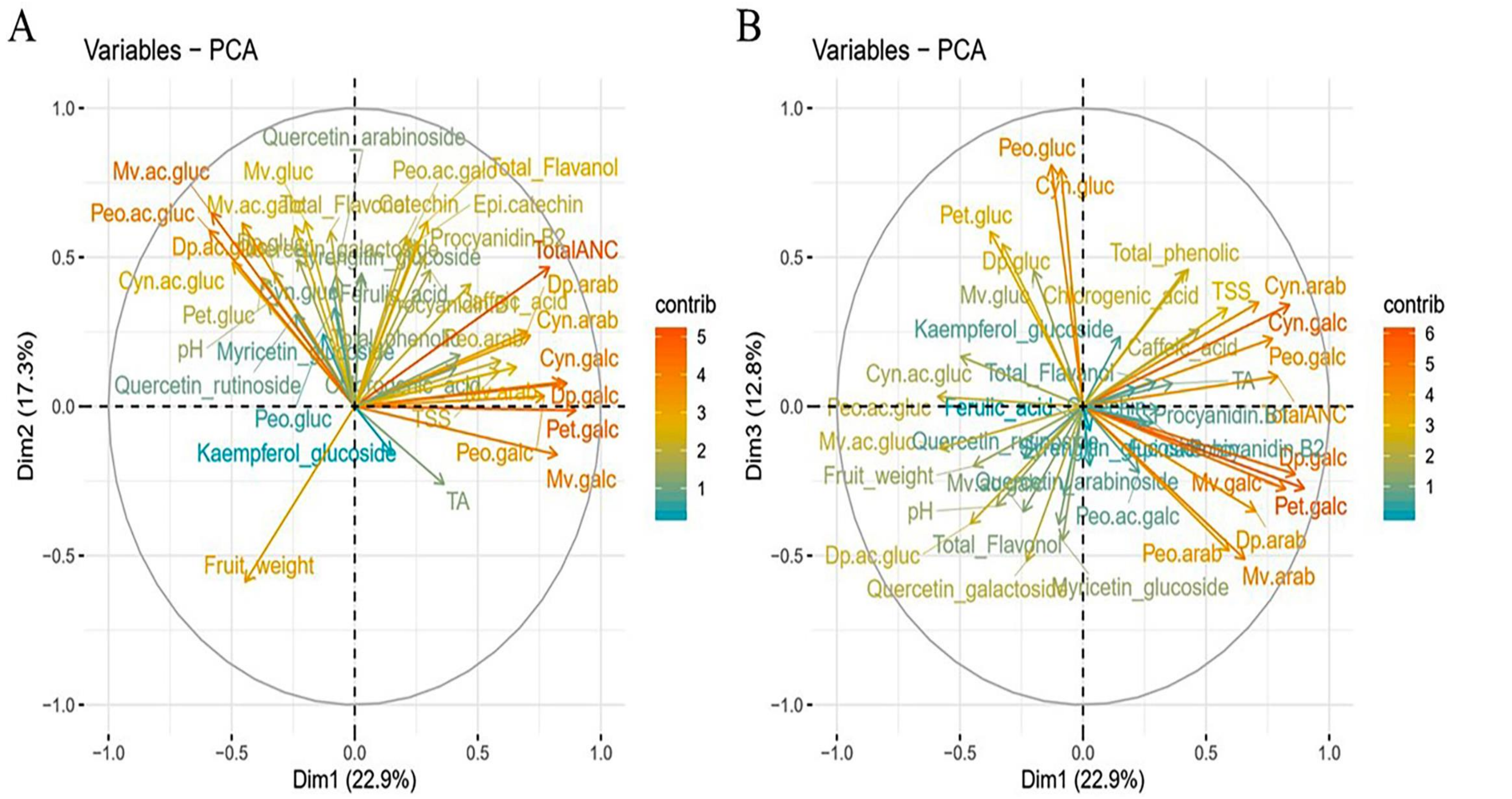

Supplementary Figure S8. PCA loading scores of the first and second (A) and the first and third (B) principal components of metabolite and fruit quality data from 128 blueberry accessions.

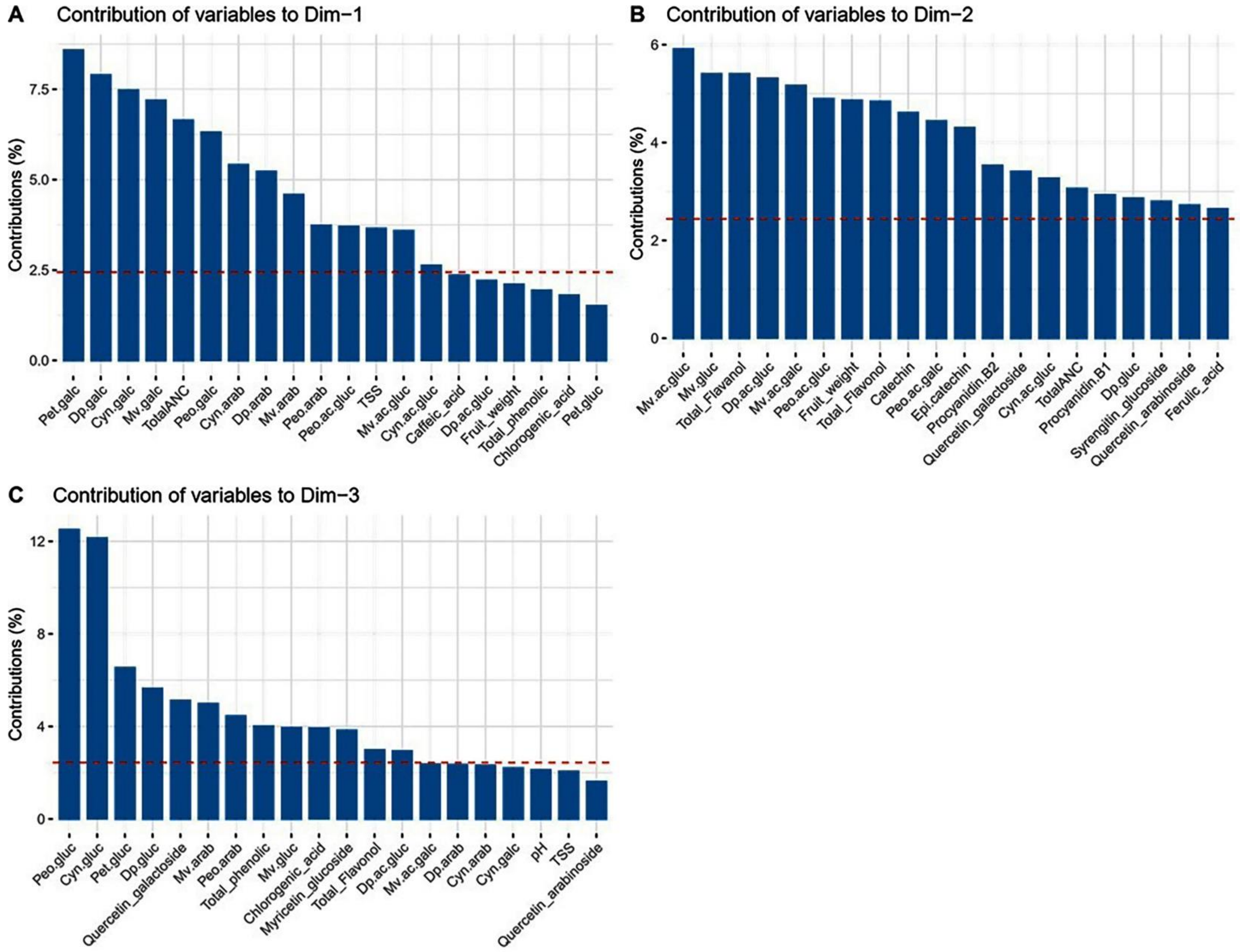

Supplementary Figure S9. Variable contribution (%) to the first (A), second (B) and third (C) principal components of the PCA of metabolite and fruit quality data from 128 blueberry accessions.

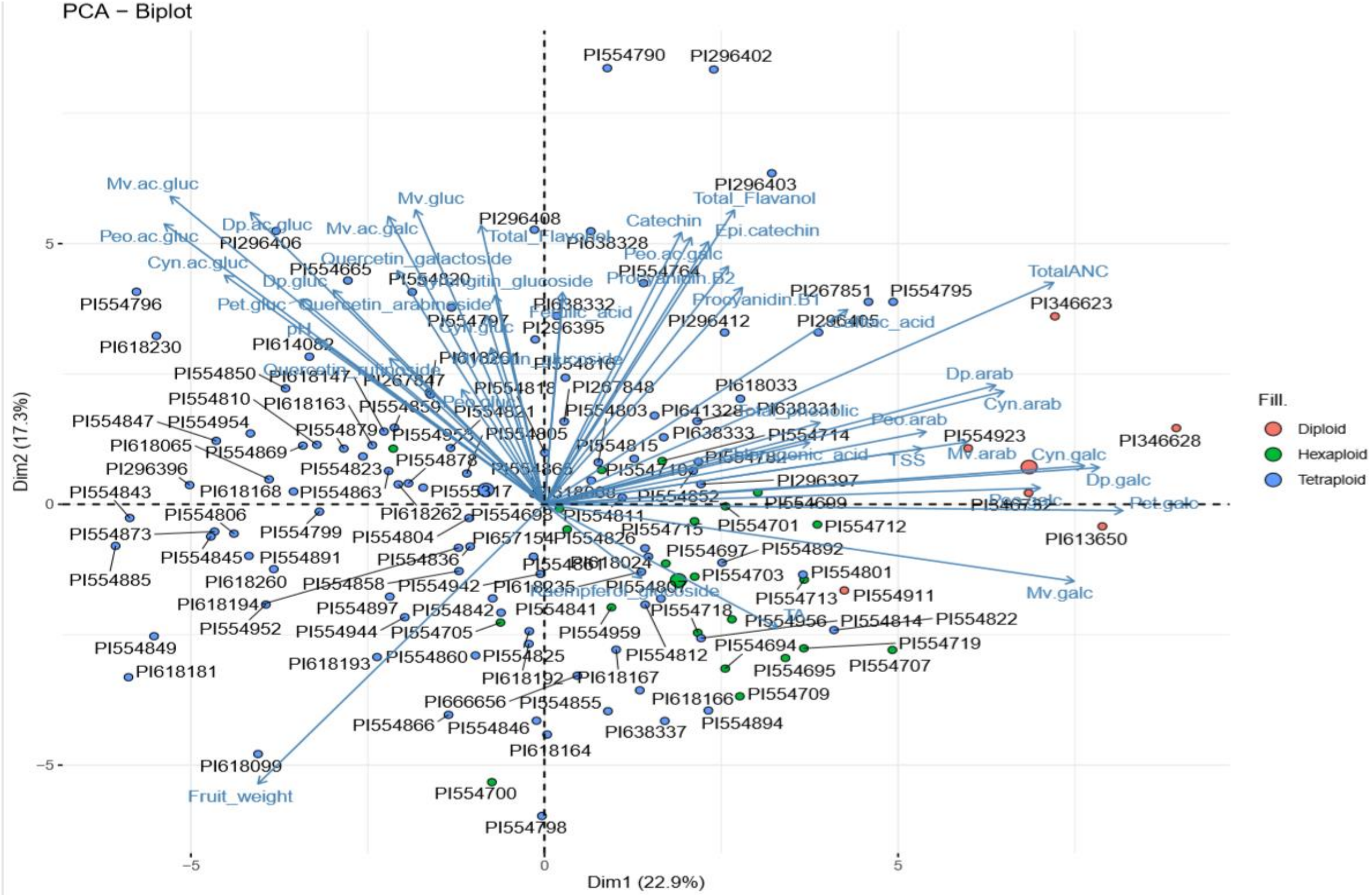

Supplementary Figure S10. PCA biplot showing the affiliation between variables and individual accessions in the first two PCA plots.

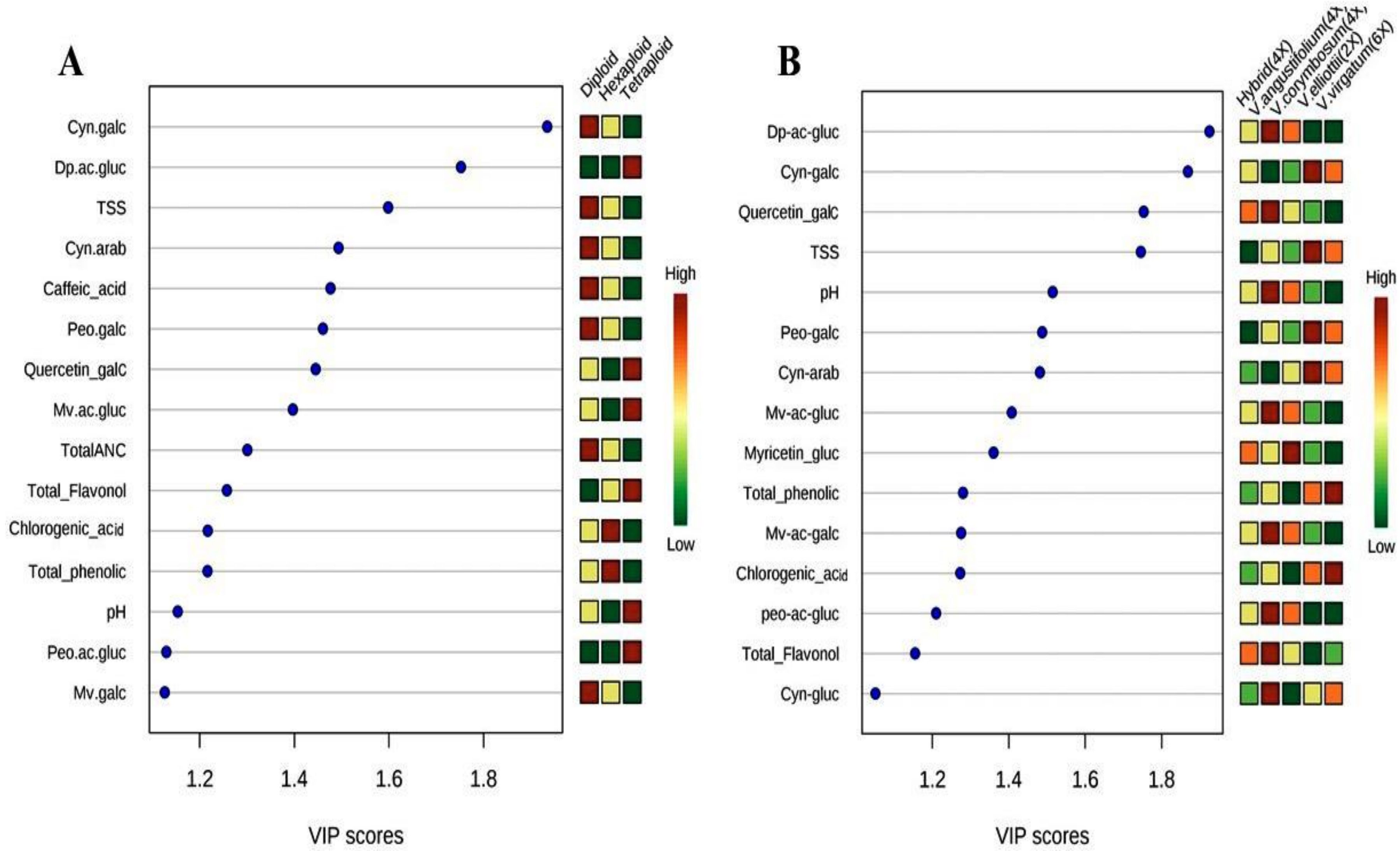

Supplementary Figure S11. Variable importance projection using PLS-DA model of metabolites and fruit quality traits of 128 blueberry accessions, ploidy-groups (A) and species levels (B).

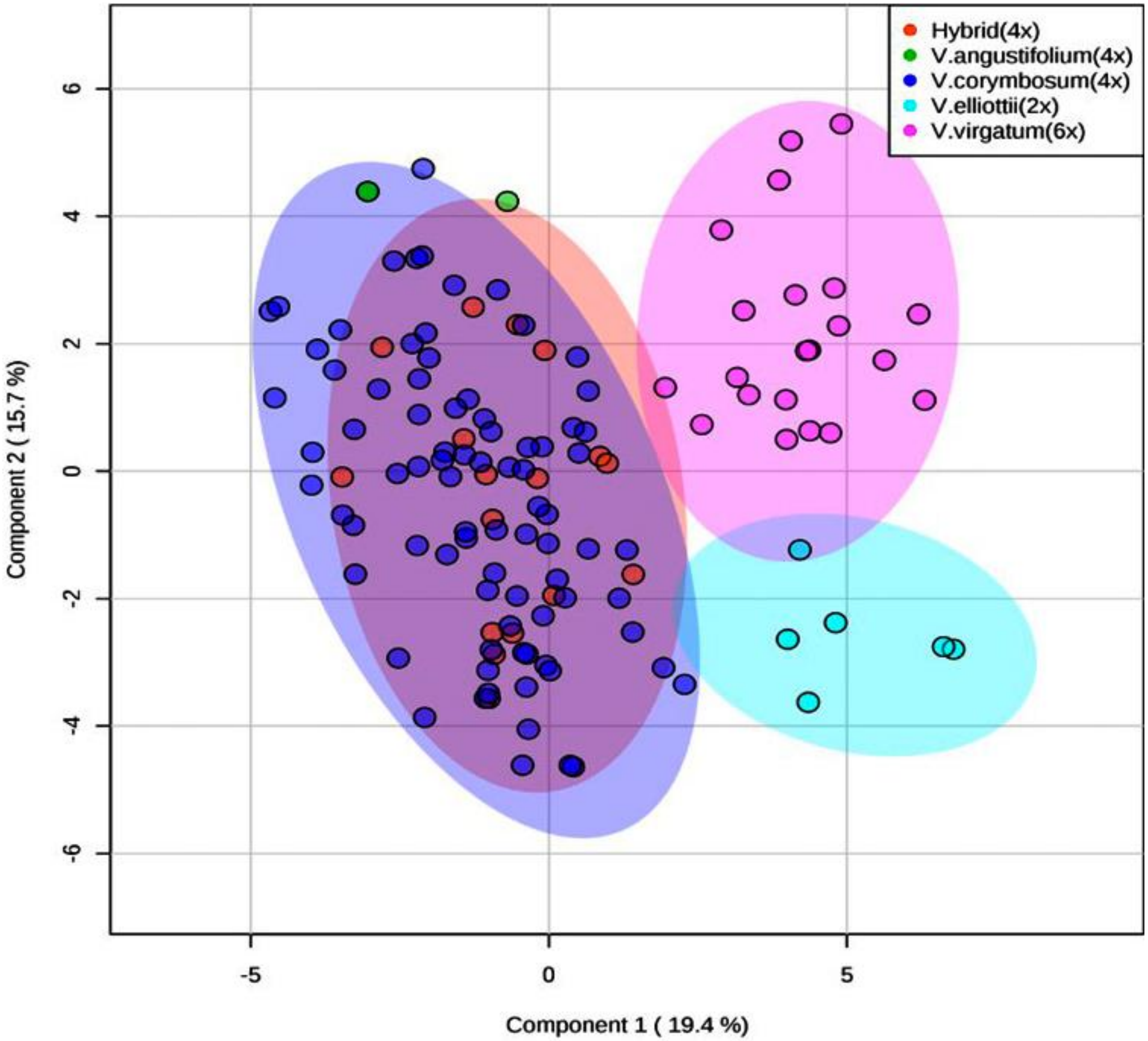

Supplementary Figure S12. PLS-DA model of metabolite and fruit quality data from 128 blueberry accessions.

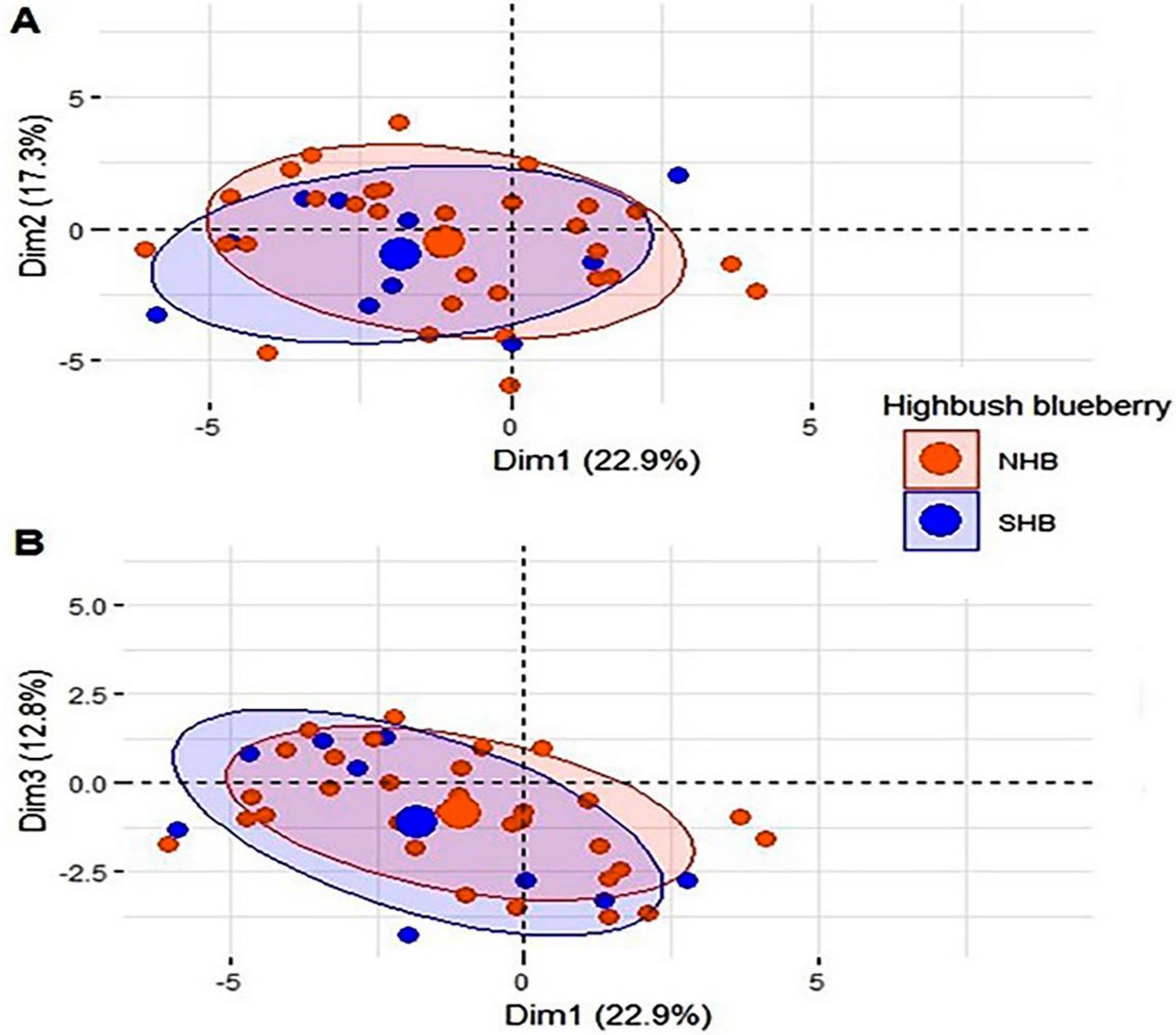

Supplementary Figure S13. PCA score plot of metabolites and fruit quality data of 128 blueberry accessions based on southern-northern highbush blueberry classification.

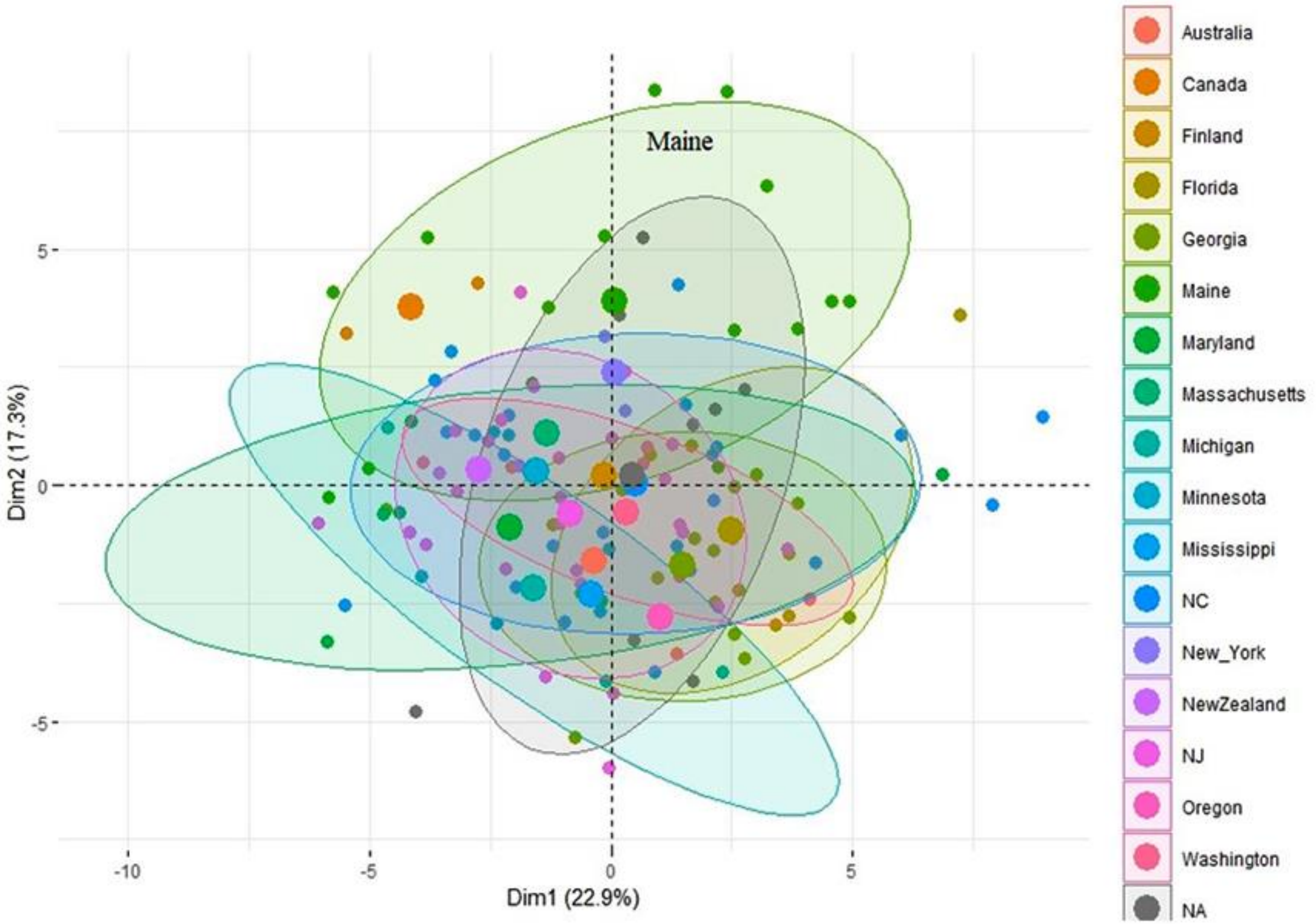

Supplementary Figure S14. PCA score plot of metabolites and fruit quality data of 128 blueberry accessions based on their sites of collection.

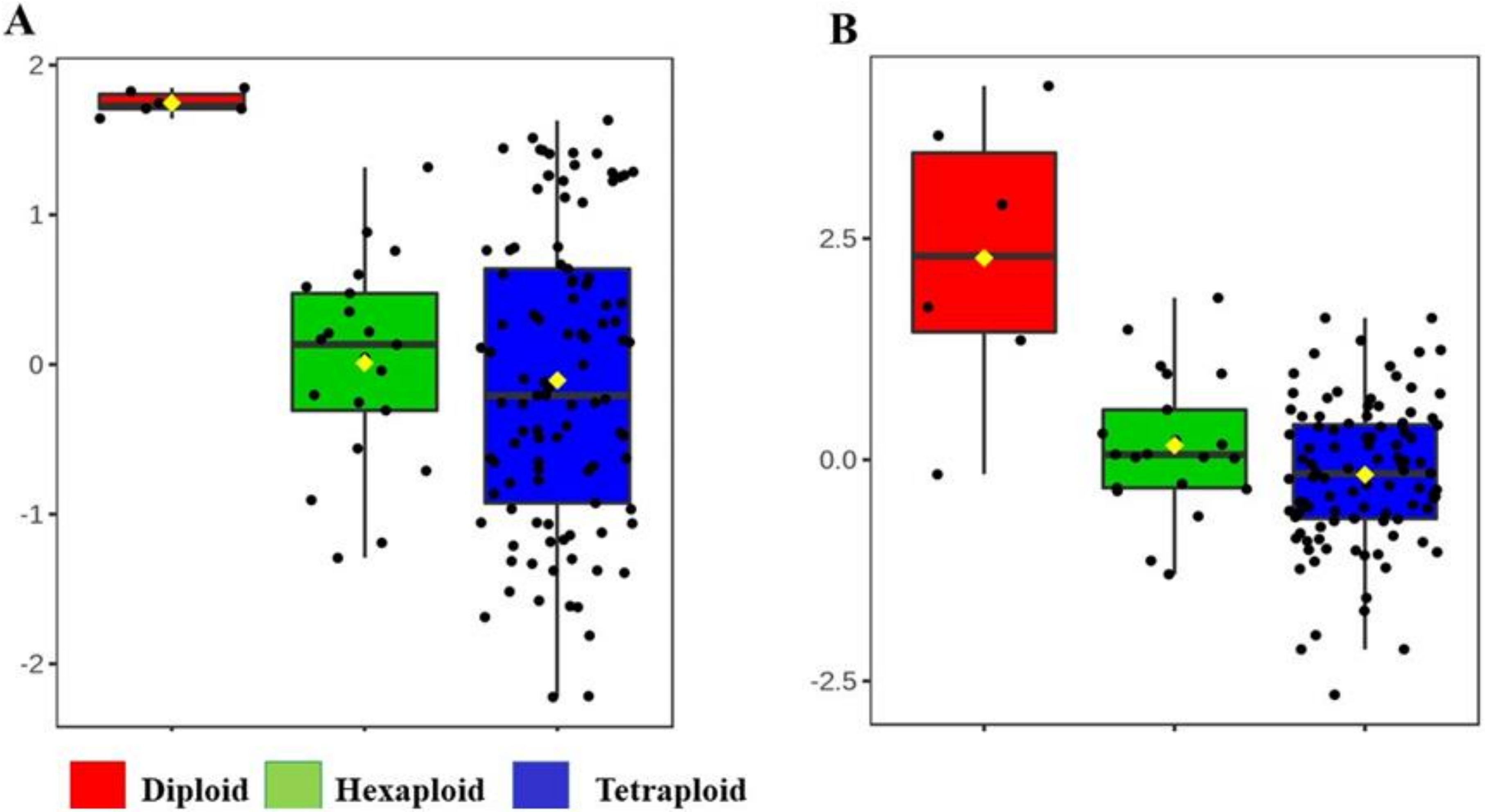

Supplementary Figure S15. Boxplot showing variation in anthocyanin concentration within and between ploidy levels of blueberry accessions. Fruit size-effect was corrected using linear regression as described by Mengist et al.(2018) and the variance component was partitioned into components associated with fruit size (A) and fruit size-corrected anthocyanin concentration (B). The data are auto scaled.
